# Association between malnutrition diagnosed by different screening and assessment tools and clinical outcomes: an umbrella review

**DOI:** 10.3389/fnut.2025.1676201

**Published:** 2025-10-09

**Authors:** Zhinan Li, Yueying Lin, Yanmei Shi, Ting Yang, Liya An, Yuxing Qi, Pengcheng Zhang, Xingzong Huang, Xianming Su, Yinlong Deng, Jian Hu, Guobin Liu, Dali Sun

**Affiliations:** ^1^Department of Gastrointestinal Surgery, Second Affiliated Hospital of Kunming Medical University/Second Faculty of Clinical Medicine, Kunming Medical University, Kunming, China; ^2^Department of Gastroenterology, Second Affiliated Hospital of Kunming Medical University/Second Faculty of Clinical Medicine, Kunming Medical University, Kunming, China

**Keywords:** clinical outcomes, malnutrition, nutritional screening, nutritional screening and assessment tools, umbrella review

## Abstract

**Background:**

Malnutrition can lead to adverse clinical outcomes in hospitalized patients, timely and accurate diagnosis of malnutrition is crucial for initiating early nutritional support programs. To assess the correlation between malnutrition diagnosed by different malnutrition diagnostic tools and patients’ clinical outcomes.

**Methods:**

Meta-analyses of the associations between malnutrition and patients’ clinical outcomes were screened and included by searching databases. For each association, this study used fixed and random effects models, calculated 95% CI (confidence intervals) and 95% PI (prediction intervals), and assessed heterogeneity, evidence of small-study effects, and excess significance bias.

**Results:**

A total of 138 meta-analyses were included in this study, and 407 associations were evaluated. For oncology patients, malnutrition diagnosed by eight tools was associated with oncological survival, with three evidence scores of PNI (prognostic nutritional index), GNRI (geriatric nutritional risk index), and CONUT (controlling nutritional status) being highly recommended (Class II). For nontumor patients, malnutrition diagnosed by nine tools was associated with poor clinical outcomes, with four tools with high evidence scores (Class II) of PNI, BMI (body mass index) < 18.5 kg/m^2^, GNRI, and CONUT being highly recommended.

**Conclusion:**

This study demonstrated a significant correlation (Class II) between malnutrition diagnosed by four tools, the PNI, BMI < 18.5 kg/m^2^, GNRI, and CONUT, and clinical outcomes, and the other tools need to be validated in future high-quality studies despite their correlation.

**Systematic review registration:**

PROSPERO CRD42024586175.

## 1 Introduction

Malnutrition is prevalent among hospitalized patients and those with chronic conditions. By 2050, the proportion of the global population aged 65 and over is projected to rise to 22%, aging may contribute to malnutrition, leading to a range of adverse clinical outcomes (1). Prompt and accurate diagnosis of malnutrition is crucial for clinical staff to identify malnourished patients and for clinicians to formulate appropriate nutritional support plans. At the same time, different malnutrition diagnosis and assessment tools not only yield varying malnutrition prevalence rates, but also cause significant confusion for clinicians and nursing staff due to differences in diagnostic accuracy (2).

The mini-nutritional assessment short-form (MNA-SF), subjective global assessment (SGA), global leadership initiative on malnutrition (GLIM), nutritional risk screening 2002 (NRS 2002), naples prognostic score (NPS), modified glasgow prognostic score (mGPS), glasgow prognostic score (GPS), mini-nutritional assessment (MNA), CONUT, GNRI, PNI, phase angle (PA), and BMI are commonly used tools for nutritional screening and assessment. Numerous meta-analyses have focused on the correlation between malnourished patients and clinical outcomes (3–79); however, since different studies have focused on different tools, the results obtained vary widely, which made it difficult for clinical specialists to obtain a comprehensive understanding of the advantages and disadvantages of different malnutrition diagnostic tools.

Therefore, by comprehensively reviewing the latest data from published systematic reviews and meta-analyses to better understand the potential bias in the correlation between different malnutrition diagnostic tools and clinical outcomes, this study provides references for clinicians and nursing staff to screen for suitable malnutrition diagnostic tools.

## 2 Methods

### 2.1 Data resources and search strategy

Systematic searches of the PubMed, Cochrane Library, Embase, ScienceDirect, Web of Science, Wanfang, CNKI, and VIP databases were conducted independently by two authors (Zhinan Li and Yueying Lin) up to October 2024. The results of the systematic searches are summarized in the following table. Disagreements were discussed with the involvement of a third researcher (Dali-Sun).

### 2.2 Study selection and extraction

Two researchers (Zhinan Li and Yueying Lin) independently screened the article titles and abstracts in October 2024, excluded irrelevant literature based on the following inclusion and exclusion criteria, according to the PICOS (Population, Intervention, Comparison, Outcome, Study design) criteria (Table 1), and independently read the full texts of eligible studies, with any inconsistencies resolved through discussion involving a third researcher (Dali-Sun). The literature inclusion criteria were as follows: (1) hospitalized adult patients; (2) patients whose nutritional status was evaluated during hospitalization via nutritional screening and assessment tools; (3) reporting correlations between nutritional risk or malnutrition and adverse clinical outcomes (e.g., survival time, mortality rate, complications, etc.); and (4) meta-analyses of observational or interventional studies. Exclusion criteria were as follows: (1) non meta-analysis; (2) not having the desired clinical outcomes (e.g., survival time, mortality rate, complications, etc.); (3) systematic reviews or meta-analyses that did not provide study-specific data; (4) meta-analyses that did not include data from studies on nutritional risk or correlation of malnutrition with poor clinical outcomes; and (5) unavailability of the original article.

**Table 1:** PICOS criteria for inclusion of studies.

Parameter	Criterion
Population	Hospitalized malnutrition patients
Interventions/exposure comparator	None
Outcome	Adverse clinical outcomes
Study design	Systematic review or meta-analyses

### 2.3 Data extraction

Data extraction was performed independently by two authors (Zhinan Li and Yueying Lin) in November 2024, with disagreements, if any, resolved through discussions involving a third researcher (Dali-Sun). We extracted the following data from each meta-analysis (Supplementary material 2): the first author, year of publication, type of study, number of studies, number of cases, study population, the age range of patients, and quality assessment tools.

### 2.4 Assessment of summary effects and heterogeneity

We analyzed the data using Stata version 17.0. For each meta-analysis, we estimated the pooled effect sizes and their 95% CI via fixed-effects and random-effects models (80, 81). After accounting for uncertainty in the pooled effects estimated in the random effects model and heterogeneity among studies, we calculated 95% PI to predict the range of expected effect sizes in the original study (82). For the largest dataset in each meta-analysis, we calculated standard error (SE) of the effect size and determined whether SE was less than 0.10. Heterogeneity between studies was assessed using the *I*^2^ statistic, and heterogeneity was considered to be significant or considerable when the *I*^2^ exceeded 50% or 75%, respectively (83).

### 2.5 Assessment of methodological quality

The methodological quality assessment of each study was carried out independently by two authors (Yanmei Shi and Guobin Liu) using the quality assessment tool “A MeaSurement Tool to Assess Systematic Reviews-2” (AMSTAR-2) (84). Disagreements were discussed with the involvement of a third researcher (Dali-Sun).

### 2.6 Assessment of small-study effects

We used the Egger’s test to determine the small-study effect, which was determined by (1) the *p* value of the Egger’s test being less than 0.10 and (2) the effect size of the largest study being smaller than the combined effect size (85).

### 2.7 Evidence of excess significance bias

P-curve and statistical standards (PSST) and effect size and significance (ESS) were introduced to calculate the expected statistically significant findings in the absence of selective reporting or publication bias, based on the mean and variance of the true distribution of effects estimated from the SE and meta-analysis of each study (86). An oversignificance test was considered positive when the *p* value was less than 0.05.

### 2.8 Sensitivity analysis

When studies with a high risk of bias and very low to low quality of evidence were excluded, the strength of evidence increased to moderate for 1 association, which was the overall survival of cholangiocarcinoma patients with malnutrition diagnosed by the CONUT score, and to high for 3 associations, which were the overall survival of patients with malnutrition and colorectal cancer diagnosed by the PNI, all-cause mortality of patients with heart failure, and hepatocellular cancer patients’ overall survival.

### 2.9 Reviewing the existing evidence

Statistically significant (*p* < 0.05) correlations between nutritional screening and assessment tools and clinical outcomes were categorized into five levels based on specific criteria (strong recommendation, high recommendation, recommendation, weak evidence, and irrelevant).

Strong recommendations included *p* < 10^−6^, number of cases > 1,000, *p* < 0.05 for the largest study in the meta-analysis, heterogeneity *I*^2^ < 50, 95% PI to exclude nulls, no small-study effect (Egger’s test *p* > 0.1), and no excessive significance bias (PSST and ESS = 0).

High recommendations included *p* < 10^−6^, number of cases >1,000, *p* < 0.05 for the largest study in the meta-analysis.

Recommendations included *p* < 10^−3^, number of cases >1,000.

For weak evidence, the only criterion was *p* < 0.05 (87).

When the *p* value is greater than 0.05, there is no association.

## 3 Results

### 3.1 Literature review and characteristics of the included articles

**Figure 1:**
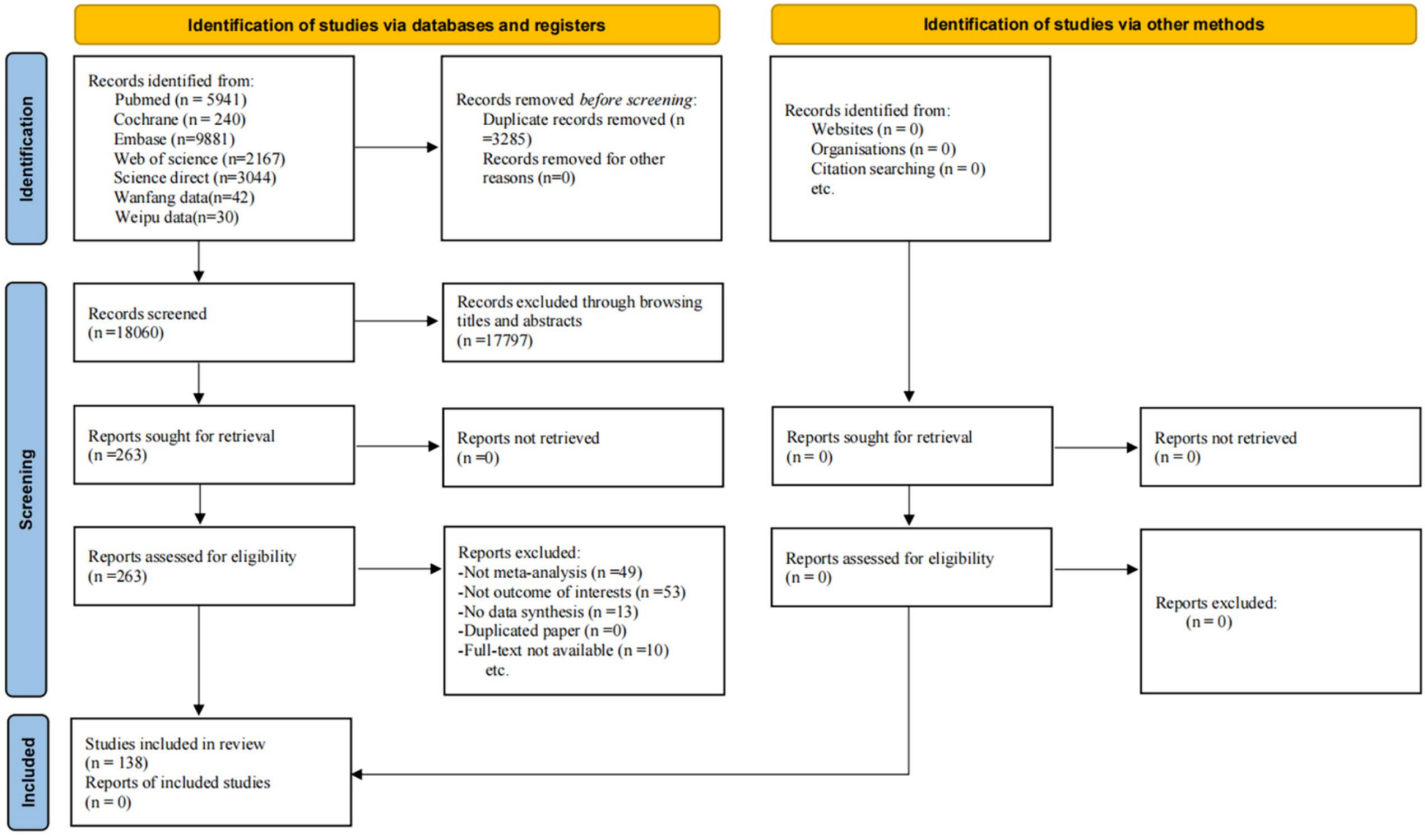
Flow diagram of the study selection process.

After a systematic search, we obtained 21,345 records from five electronic databases. After removing duplicates, 17,797 records were excluded by browsing titles and abstracts, and 125 records were excluded after the full texts were assessed. Ultimately, 138 studies met our inclusion criteria and were included in the final analysis (Figure 1). The characteristics of the included studies are shown in Supplementary material 2.

Methodological quality was assessed for 138 studies using AMSTAR-2. The descriptive characteristics of the included meta-analyses by type of nutritional screening and assessment tools are summarized in Table 2. The studies included 407 associations between different nutritional screening and assessment tools and clinical outcomes, and all the articles were published in 2012–2024, with sample sizes ranging from 187 to 308,430 cases.

**Table 2:** Descriptive statistics for the overall meta-analysis and the types of nutritional screening and assessment tools included in the umbrella review that graded nutritional screening and assessment tools against evidence of clinical outcomes.

	Total	MNA-SF	SGA	GLIM	NRS 2002	NPS	MGPS	GPS	MNA	CONUT	GNRI	PNI	PA	BMI
Number of meta-analyses	2,233	93	31	55	33	20	63	11	52	409	407	827	35	197
Number of studies	158	4	1	5	2	2	4	1	3	31	34	63	3	5
Median	4–62	11–38	31	7–15	11–22	7–13	11–25	11	10–31	5–62	6–38	6–42	4–20	22–61
Min-max Number of cases	253,460	15,991	25,141	45,563	6,430	4,489	3,074	2,830	11,378	7,557	6,267	6,050	3,217	115,413
Median	187–308,430	4,300–30,043	25,141	3,662–14,573	3,527–9,332	1,657–7,321	2,391–4,629	2,830	4,300–25,141	1,220–36,198	1,354–30,043	187–30,043	2,625–3,770	21,150–308,430
Min-max														

BMI, body mass index; CONUT, controlling nutritional status; GLIM, global leadership initiative on malnutrition; GNRI, geriatric nutritional risk index; GPS, Glasgow Prognostic Score; mGPS, modified Glasgow Prognostic Score; MNA, mini-nutritional assessment; MNA-SF, mini-nutritional assessment short-form; NPS: naples prognostic score; NRS 2002, nutritional risk screening 2002; PA, phase angle; PNI, prognostic nutritional index; SGA, subjective global assessment.

### 3.2 Summary effect size

A meta-analysis of 407 associations was conducted using random and fixed effects models. Among these, 161 (39.6%) meta-analyses had statistically significant pooled random and fixed effects estimates at *p* ≤ 0.05. Ninety-four (23.1%) meta-analyses showed significant results when the more stringent *p* ≤ 10^−3^ was used as the significance threshold, whereas 85 (20.9%) meta-analyses remained significant at a threshold of 10^−6^ (Table 3). Approximately half (42.5%) of the meta-analyses were highly heterogeneity, with the largest proportion of highly heterogeneity meta-analyses of MNA with clinical outcomes (66.7%). SGA and GPS did not have highly heterogeneity meta-analyses with clinical outcomes, followed by BMI (61.5%), NPS (60%), GLIM (44.8%), PNI (43.8%), CONUT (41.3%), and GNRI (41.3%) (Table 3). The proportion of meta-analyses with little evidence of heterogeneity (*I*^2^ ≤ 25%) was 39.6%. There were 396 (97.3%) associations with 95% PI excluding the null (Table 3).

**Table 3:** Number and percentage of meta-analyses that met the individual and overall criteria for individual and overall and nutritional screening and assessment tool types used for nutritional screening and assessment tools and clinical outcomes.

	Total	MNA-SF	SGA	GLIM	NRS 2002	NPS	MGPS	GPS	MNA	CONUT	GNRI	PNI	PA	BMI
Criterion														
*p* value < 10^−6^, *n* (%)	85 (20.9)	1 (25)	0 (0)	6 (20.7)	2 (33.3)	1 (20)	2 (22.2)	0 (0)	1 (33.3)	14 (17.5)	15 (18.8)	41 (24.3)	0 (0)	2 (15.4)
*p* value < 10^−3^, *n* (%)	94 (23.1)	1 (25)	0 (0)	6 (20.7)	2 (33.3)	2 (40)	2 (22.2)	0 (0)	1 (33.3)	16 (20)	16 (20)	46 (27.2)	0 (0)	2 (15.4)
*p* value < 0.05, *n* (%)	161 (39.6)	1 (25)	0 (0)	9 (31)	2 (33.3)	3 (60)	3 (33.3)	0 (0)	2 (66.7)	31 (38.8)	31 (38.8)	71 (42.0)	1 (16.7)	7 (53.8)
*I*^2^ > 50%, *n* (%)	173 (42.5)	1 (25)	0 (0)	13 (44.8)	2 (33.3)	3 (60)	3 (33.3)	0 (0)	2 (66.7)	33 (41.3)	33 (41.3)	74 (43.8)	1 (16.7)	8 (61.5)
*I*^2^ ≤ 25%, *n* (%)	161 (39.6)	3 (75)	1 (100)	9 (31)	4 (66.7)	0 (0)	4 (44.4)	0 (0)	1 (33.3)	33 (41.3)	34 (42.5)	67 (39.6)	3 (50)	2 (15.4)
Prediction interval excluding the null, *n* (%)	396 (97.3)	4 (100)	1 (100)	29 (100)	5 (83.3)	5 (100)	9 (100)	2 (100)	3 (100)	78 (97.5)	79 (98.8)	164 (97.0)	4 (66.7)	13 (100)
Evidence of small study bias, *n* (%)	199 (48.9)	3 (75)	0 (0)	12 (41.4)	2 (33.3)	2 (40)	2 (22.2)	1 (50)	0 (0)	45 (56.3)	51 (63.8)	69 (40.8)	4 (66.7)	8 (61.5)
Evidence of excess significance bias, *n* (%)	160 (39.3)	0 (0)	0 (0)	10 (34.5)	1 (16.7)	2 (40)	3 (33.3)	1 (50)	2 (66.7)	34 (42.5)	37 (46.3)	65 (38.5)	0 (0)	5 (38.5)
Overall grading														
Not significant, *n* (%)	246 (60.4)	3 (75)	1 (100)	21 (72.4)	4 (66.7)	2 (40)	6 (66.7)	2 (100)	1 (33.3)	49 (61.2)	49 (61.2)	97 (57.4)	5 (83.3)	6 (46.1)
Weak, *n* (%)	67 (16.5)	0 (0)	0 (0)	2 (6.9)	0 (0)	1 (20)	1 (11.1)	0 (0)	1 (33.3)	15 (18.8)	15 (18.8)	26 (15.4)	1 (16.7)	5 (38.5)
Suggestive, *n* (%)	61 (15.0)	1 (25)	0 (0)	6 (20.7)	2 (33.3)	2 (40)	2 (22.2)	0 (0)	1 (33.3)	12 (15)	8 (10)	26 (15.4)	0 (0)	1 (7.7)
Highly suggestive, *n* (%)	33 (8.1)	0 (0)	0 (0)	0 (0)	0 (0)	0 (0)	0 (0)	0 (0)	0 (0)	4 (5)	8 (10)	20 (11.8)	0 (0)	1 (7.7)
Strong, *n* (%)	0 (0)	0 (0)	0 (0)	0 (0)	0 (0)	0 (0)	0 (0)	0 (0)	0 (0)	0 (0)	0 (0)	0 (0)	0 (0)	0 (0)

BMI, body mass index; CONUT, controlling nutritional status; GLIM, global leadership initiative on malnutrition; GNRI, geriatric nutritional risk index; GPS, Glasgow Prognostic Score; mGPS, modified Glasgow Prognostic Score; MNA, mini-nutritional assessment; MNA-SF, mini-nutritional assessment short-form; NPS: naples prognostic score; NRS 2002, nutritional risk screening 2002; PA, phase angle; PNI, prognostic nutritional index; SGA, subjective global assessment.

### 3.3 Small-study effects

According to the Egger’s test (*p* < 0. 10), 199 (48.9%) meta-analyses showed evidence of low study effect, with the highest bias for the MNA-SF score and patient clinical outcomes (75%; only 1 meta-analyses available), followed by PA (66.7%), BMI (61.5%), GNRI (63.8%), CONUT (56.3%) and GPS (50%) (Table 3).

### 3.4 Excess significance

The proportion of meta-analyses showing evidence of excess significance bias was 39.3%, ranging from 0% for the MNA-SF, SGA, and PA to 66.7% for the MNA (Table 3), and the highest correlations were between the MNA and overall survival (33.3%) in older cancer patients and all-cause mortality (33.3%) in patients with heart failure (Supplementary material 4 and Table 3).

### 3.5 Grading of the evidence

**Figure 2:**
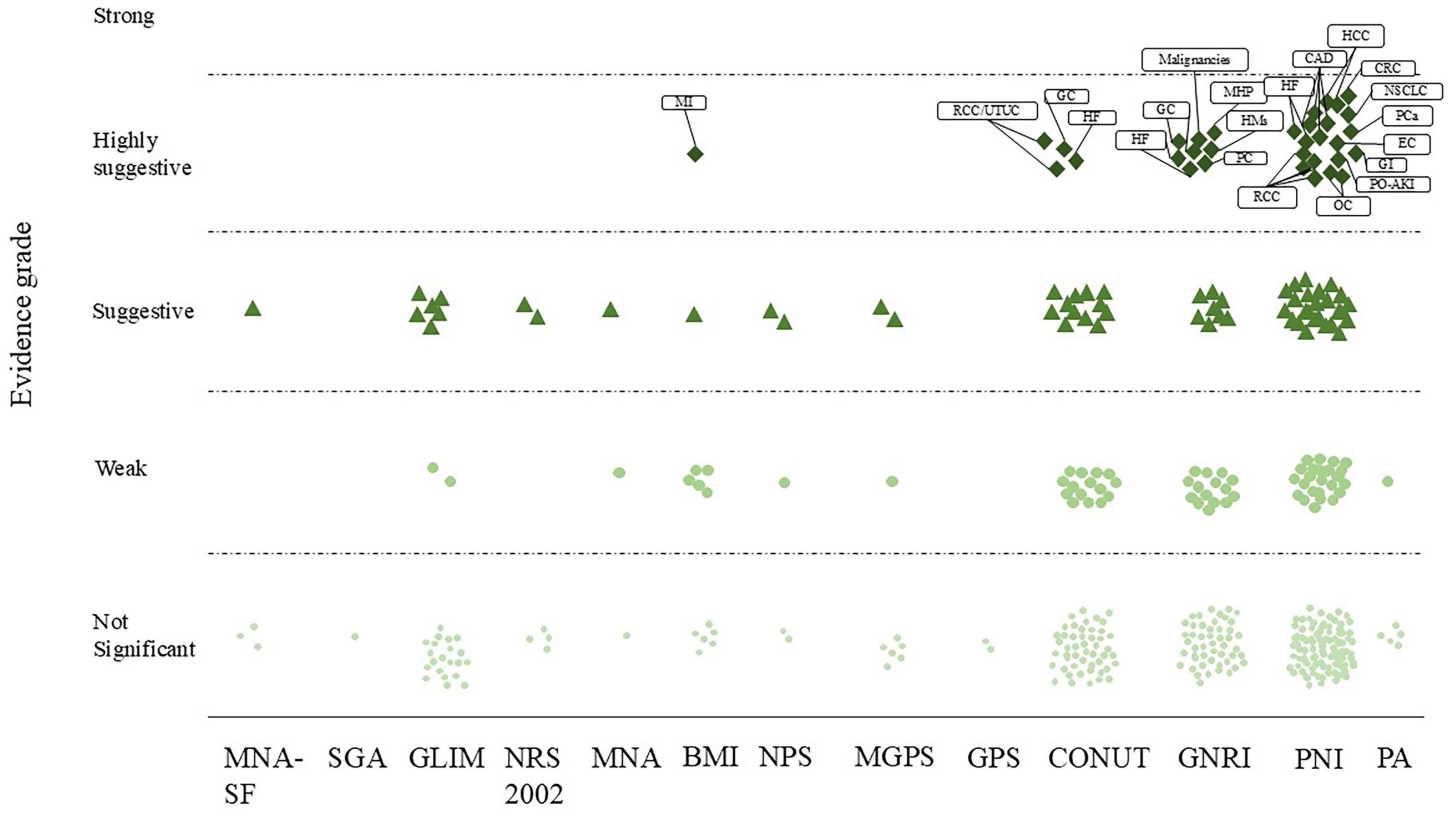
Scatterplot showing the results of an umbrella review grading nutritional screening and assessment tools against clinical outcomes. The y-axis shows the strength of the evidence. The x-axis corresponds to different nutritional screening and assessment tools. CAD, coronary artery disease; CRC, colorectal cancer; EC, esophageal cancer; GI, gynecological cancer; GC, gastric cancer; HCC, hepatocellular carcinoma; HF, heart failure; HMs, hematologic malignancies; MHP: maintenance hemodialysis patients; MI, myocardial infarction; NSCLC, non-small cell lung cancer; OC, ovarian cancer; PC, pancreatic cancer; PCa, prostate cancer; PO-AKI, postoperative acute kidney injury risk; RCC, renal cell carcinoma; UTUC, upper tract urothelial carcinoma.

Thirty-three meta-analyses (8.1%) provided high-level evidence (Figures 1, 2 and Supplementary materials 3, 4), mainly concerning the PNI (*n* = 20), followed by the GNRI (*n* = 8), CONUT score (*n* = 4) and BMI (*n* = 1). PNI-diagnosed malnutrition was strongly associated with overall survival in patients with non-small cell lung, colorectal, hepatocellular, esophageal, renal cell, prostate and gynecologic malignancies; mortality and major adverse cardiovascular events (MACEs) in patients with coronary artery disease; and the risk of postoperative acute kidney injury (PO-AKI). Malnutrition diagnosed by the GNRI was strongly associated with overall survival in patients with gastric cancer, hematologic malignancies, and undifferentiated malignancies and mortality in patients with heart failure, patients after transcatheter aortic valve implantation (TAVI), and those receiving hemodialysis. The CONUT score was strongly associated with the risk of complications in patients with gastric cancer, overall survival in patients with upper urinary tract uroepithelial or renal cell carcinoma and in patients with undifferentiated cancer, and all-cause mortality in patients with heart failure. Underweight (BMI < 18.5 kg/m^2^) was associated with long-term mortality after myocardial infarction. Sixty-one (15.0%) meta-analyses provided suggestive evidence that malnutrition diagnosed by the MNA-SF was strongly associated with mortality in patients after hip fracture surgery. Malnutrition diagnosed by the GLIM criteria was strongly associated with the overall survival of cancer patients, complications in patients with hepatopancreatobiliary and gastric cancers, and patients with undifferentiated cancers. Malnutrition, as determined by the NRS 2002, was associated with the length of hospitalization in patients undergoing abdominal surgery and overall survival in cancer patients. An NPS-based diagnosis of malnutrition is associated with overall survival in patients with gastrointestinal and lung cancers. The presence of malnutrition according to the mGPS was strongly associated with overall survival in patients with pancreatitis and cholangiocarcinoma. A diagnosis of malnutrition via the MNA was associated with overall survival in elderly cancer patients. A CONUT score indicating malnutrition was associated with overall survival in patients with pancreatic cancer, cholangiocarcinoma, lymphoma, and undifferentiated malignancies; complications in patients with gastric, hepatopancreatobiliary, and undifferentiated malignancies; mortality in patients with decompensated heart failure and after TAVI; and MACEs in patients with coronary artery disease. GNRI-diagnosed malnutrition was associated with survival in patients with diffuse large B-cell lymphoma, urologic cancers, pancreatic cancer, and undifferentiated hematologic malignancies; mortality in patients hospitalized for postoperative hip fracture and decompensated heart failure; and major cardiovascular events in elderly patients with heart failure (RR, 2.00; 95% CI, 1.24–3.22). PNI-diagnosed malnutrition was associated with survival in patients with lung, oral cavity, esophageal, gastric, colorectal, renal, breast, ovarian malignancies, undifferentiated gynecologic malignancies, glioma and patients treated with immune checkpoint inhibitor; mortality in patients with heart failure and acute coronary syndromes; and the risk of intensive care unit admission for PO-AKI (MD, 0.98; 95% CI, 0.15–1.81). In addition, underweight (BMI < 18.5 kg/m^2^) was associated with overall survival in patients after colorectal cancer surgery. A total of 67 meta-analyses (16.5%) were supported by weak evidence, and the remaining 246 (60.4%) meta-analyses had no statistically significant results.

## 4 Discussion

In order to provide a comprehensive understanding of the strength of the association between malnutrition diagnosed by different nutritional screening and assessment tools and adverse clinical outcomes in patients, we systematically evaluated 138 meta-analyses and 13 different nutritional screening and assessment tools. Thirty-three associations were supported by high-level recommendations, including that PNI-diagnosed malnutrition is associated with survival in oncology patients with non-small cell lung cancer, colorectal cancer, hepatocellular carcinoma, esophageal cancer, renal cell carcinoma, prostate cancer, and gynecologic malignancies; mortality and MACEs in patients with coronary artery disease; and patients’ risk of PO-AKI, which are closely related to adverse clinical outcomes (88–100); underweight (BMI < 18.5 kg/m^2^) and long-term mortality after myocardial infarction in patients (101); GNRI-diagnosed malnutrition is strongly associated with survival in patients with gastric cancer, hematologic malignancies, and undifferentiated malignancies; and mortality in patients with heart failure, patients after TAVI, and hemodialysis patients (20, 91, 102–106). CONUT score-diagnosed malnutrition is strongly associated with the risk of complications in patients with gastric cancer, survival in patients with upper urinary tract uroepithelial or renal cell carcinoma and in patients with undifferentiated cancer, and all-cause mortality in patients with heart failure (90, 107). Sixty-one meta-analyses provided suggestive evidence that MNA-SF-diagnosed malnutrition is strongly associated with mortality in patients after hip fracture surgery (108). GLIM-diagnosed malnutrition is strongly associated with overall survival of cancer patients, complications in patients with esophageal, gastric cancers and patients with undifferentiated cancers (108–110). Malnutrition diagnosed by the NRS 2002 is strongly associated with length of hospital stay in patients who underwent abdominal surgery and cancer patients’ overall survival (38, 111). NPS-diagnosed malnutrition is associated with survival in patients with gastrointestinal and lung cancers (112, 113). MGPS-diagnosed malnutrition is strongly associated with survival in patients with pancreatic and bile duct cancers (114, 115). MNA-diagnosed malnutrition is associated with survival in elderly cancer patients (116). CONUT-diagnosed malnutrition is associated with survival in patients with pancreatic cancer, cholangiocarcinoma, lymphoma, and undifferentiated malignancies, complications in patients with gastric cancer, hepatopancreaticobiliary, and undifferentiated malignancies, mortality in patients after TAVI, and MACEs in patients with coronary heart disease (105, 117–124). GNRI-diagnosed malnutrition is associated with survival in patients with diffuse large B-cell lymphoma, urologic cancers, pancreatic cancer, and undifferentiated hematologic malignancies, mortality in patients hospitalized for postoperative hip fracture and decompensated heart failure, and major cardiovascular events in elderly patients with heart failure (106, 108, 121, 125–128). PNI-diagnosed malnutrition is associated with survival in patients with lung, oral cavity, esophageal, gastric, colorectal, renal, breast, ovarian, undifferentiated gynecologic malignancies, glioma, and patients treated with immune checkpoint inhibitors, mortality in patients with heart failure and acute coronary syndromes, and the risk of intensive care unit admission for patients with PO-AKI (46, 75, 88, 94, 96, 97, 100, 129–141). Underweight (BMI < 18.5 kg/m^2^) is associated with colorectal cancer overall survival in postoperative patients (142).

Malnutrition diagnosed by eight tools is associated with tumor survival, with three evidence scores of PNI, GNRI, and CONUT being highly recommended, followed by five evidence scores of GLIM, NRS 2002, NPS, mGPS, and MNA being recommended, which were analyzed for the following reasons: (1) the tools used assessment indicators with different focuses, and the five tools, PNI, CONUT, GNRI, NPS, mGPS, used serum albumin levels, which have previously been regarded as indicators of nutritional status (143, 144); however, it is now considered that serum albumin levels reflect the ability of the body’s liver to synthesize albumin. In patients with malignant tumors, the relatively insufficient intake of substrates for protein synthesis by the liver is caused by the high consumption of tumors, leading to decreased gastrointestinal intake ability due to the side effects of radiotherapy; on the other hand, it may be caused by the poor synthesis function of the liver due to liver tumors, drug-induced liver injuries, and other factors. Therefore, the albumin level can reflect the survival of patients with tumors to a certain extent (144). The four tools, the PNI, GNRI, GLIM and mGPS, add immune indices as indicators of malnutrition; the PNI and GNRI use lymphocyte counts, and the GLIM and mGPS use C-reactive protein (CRP). Malignant tumors are often considered to be caused by immune deficiency, and lymphocyte counts represent, to a certain degree, the body’s immune status. CRP is part of the nonspecific immune mechanism of the body and is often used to represent the level of inflammation in the body. GLIM, NRS 2002, and MNA include changes in eating status and weight/BMI, and the eating status is related to whether the tumor site is involved in the digestive system. At the same time, the gastrointestinal side effects of radiation and chemotherapy and inflammation caused by tumors can also lead to a decrease in appetite and inadequate food intake. A decrease in weight/BMI is caused by malignant tumor consumption in the body and insufficient food intake in the body, which are related to the rate of tumor progression, and often, a significant short-term decrease in weight/BMI predicts a poor clinical outcome. (2) Tools for different populations, such as the GNRI and MNA, are malnutrition diagnostic tools for elderly individuals. In addition to age as an important factor, the MNA takes into account the mental and psychological status and the activities and physical function of the nutritional status of elderly individuals, but in elderly patients with malignant tumors, other nontumor diseases, such as cardio-cerebrovascular disease and diabetes, has a greater impact on survival. (3) Differences in the sensitivity of tools to diagnose malnutrition in different tumors, for example, digestive malignancies and changes in eating status, and tools covering eating status, such as the MNA, NRS 2002 and GLIM, are more likely to identify malnourished patients, whereas eating status can clearly affect the survival of patients with tumors.

Malnutrition diagnosed with four tools, PNI, BMI < 18.5 kg/m^2^, GNRI, and CONUT, is associated with nononcologic disease adverse clinical outcomes, and the tools are highly recommended. The five tools, PNI, GNRI, CONUT, MNA-SF, and NRS 2002-diagnosed malnutrition associated with nononcologic disease adverse clinical outcomes are recommended, and the reasons for this are as follows: (1) The impact of the disease site on tool evaluation and adverse clinical outcomes of nononcologic diseases mainly include mortality from coronary heart disease, mortality from myocardial infarction, mortality from heart failure, mortality from patients after TAVI, adverse cardiovascular events, mortality from patients on hemodialysis, mortality from patients after hip fracture surgery, PO-AKI, postoperative length of stay and complications. For example, tools that include activity and physical function (e.g., MNA-SF) are more likely to identify malnourished patients with hip fracture, whereas in most patients with cardiac-related diseases, changes in cardiac function leading to fluid retention and increased vascular permeability can lead to a decrease in plasma albumin, which is more pronounced if there is also a lack of hepatic synthesis, so plasma albumin is important as an indicator to evaluate the nutritional status (e.g., PNI, GNRI, CONUT, etc.). (2) The relationships between the indicators of adverse clinical outcomes and nutritional status varies; for nononcologic diseases, malnutrition status tends to be closely related to the indicators of infectious complications and length of hospitalization, whereas there is no significant relationship with noninfectious complications (145). In the present study, malnutrition in the NRS 2002 diagnosis and treatment and length of hospitalization of patients undergoing abdominal surgery were closely related. However, the adverse clinical outcomes of nononcologic diseases were mainly mortality from cardiovascular diseases, which are closely related to adverse cardiovascular events. Although the application of these tools (PNI, BMI < 18.5 kg/m^2^, GNRI, CONUT) can predict adverse clinical outcomes in patients with cardiovascular diseases, it should not be assumed that these adverse clinical outcomes are closely related to malnutrition.

According to the principles of the WHO’s disease screening, the main criterion for validating any screening tool or diagnostic method is that the available treatment will improve the clinical outcome in a group of patients who test positive for that screening (146). As a malnutrition diagnostic tool, we can consider a malnutrition diagnostic tool valid if it can diagnose the malnutrition status of patients in a timely manner, advocate for adequate nutritional support, and improve the clinical outcome of patients.

This study involves 13 diagnostic tools for malnutrition, the results of which include clinical manifestations, signs, body fluids and blood. Some clinical manifestations, such as digestive symptoms, eating status, activity and physical functioning, the disease itself, and changes in weight/BMI, are often the reasons for a patient’s visit to the clinic, and administering the diagnostic tool at the time of the patient’s hospital admission offers the early opportunity for clinicians to detect the beginnings of malnutrition. Malnutrition diagnostic tools (GLIM, NRS 2002, MNA, SGA, MNA-SF, and BMI) that use clinical signs as indicators may offer a better chance of detecting a patient’s malnutrition status. Fluid and blood indicators are used in other malnutrition diagnostic tools, such as NPS, mGPS, GPS, CONUT, and PNI. Changes in fluid and blood indicators tend to lag behind changes in clinical indicators, and thus, these diagnostic tools tend to lag behind in identifying malnutrition status, although they may be more accurate. With the delay, the prognosis of improving clinical outcomes is poor, even with adequate nutritional support. The clinical outcome of such patients is also poor. Moreover, changes in body fluid and blood indices are strongly influenced by the state of the body; for example, when acute infection occurs, the serum albumin and lymphocyte counts are significantly reduced, and the CRP level is significantly increased. When the infection is controlled, these indices return to normal in a short period of time, and evaluating nutritional status in such a state is inappropriate. Therefore, when selecting body fluid and blood indicators as diagnostic markers for malnutrition, it is necessary to exclude disease states such as acute infection and septic shock, while also requiring repeated monitoring. Instead, evaluation tools that combine clinical manifestations and signs with blood indicators (e.g., GLIM) are recommended due to their ability to balance early detection and diagnostic sensitivity. In this study, only evidence for a recommendation of GLIM was found in relation to the overall survival of cancer patients, and considering that GLIM was released in 2019, most recently among the 13 tools, there is a lack of adequate research for further validation.

The strengths of this study are as follows: (1) This is the first umbrella review to comprehensively explore the correlation between malnutrition diagnostic tools and adverse clinical outcomes. (2) Thirteen malnutrition diagnostic tools currently in use internationally were analyzed, and malnutrition diagnostic tools with a high level of evidence were recommended for use by clinical experts. The limitations of this study include the following: (1) The early or late development of tools has a great impact on the amount of evidence, resulting in the lack of sufficient high-quality evidence for some malnutrition diagnostic tools of recent years, such as the GLIM and the NPS, which are newer tools released in 2019 and 2017, respectively. The lack of sufficient evidence for these tools does not mean the tools themselves are ineffective. (2) Owing to the ethical considerations in clinical research, most of the clinical studies in recent years were unable to use blank control; therefore, the results from the meta-analysis included in this study could not completely exclude the effect of nutritional support on clinical outcomes. (3) Some of the tools were designed for different target populations, such as the MNA and GNRI, which were designed specifically for elderly individuals, and the NPS was designed for oncology patients, which resulted in the validation of the tools only in the target populations and could not be involved in the comparison of validity in other population.

**Figure 3:**
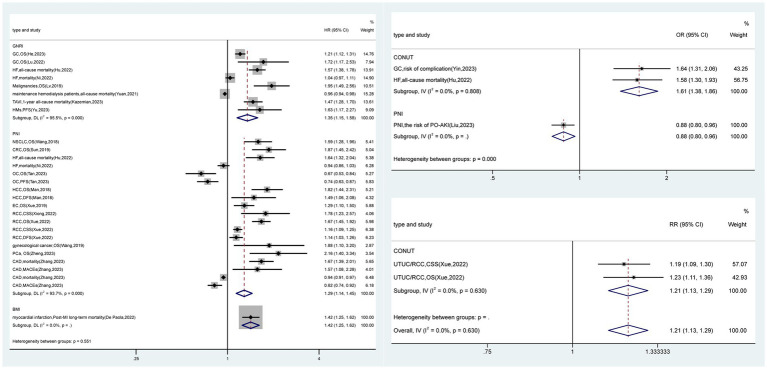
The forest plot shows the results of highly suggestive evidence from an umbrella review of nutritional screening and assessment tools and clinical outcomes. Data are expressed as hazard/risk/odds ratios and 95% confidence intervals. CAD, coronary artery disease; CRC, colorectal cancer; CSS, cancer-specific survival; DFS, disease free survival; EC, esophageal cancer; GC, gastric cancer; HCC, hepatocellular carcinoma; HF, heart failure; HMs, hematologic malignancies; MACEs, major adverse cardiovascular events; NSCLC, non-small cell lung cancer; OC, ovarian cancer; OS, overall survival; PCa, prostate cancer; PFS, progression-free survival; PO-AKI, postoperative acute kidney injury; RCC, renal cell carcinoma; TAVI, patients who underwent transcatheter aortic valve implantation; UTUC, upper tract urothelial carcinoma.

**Figure 4:**
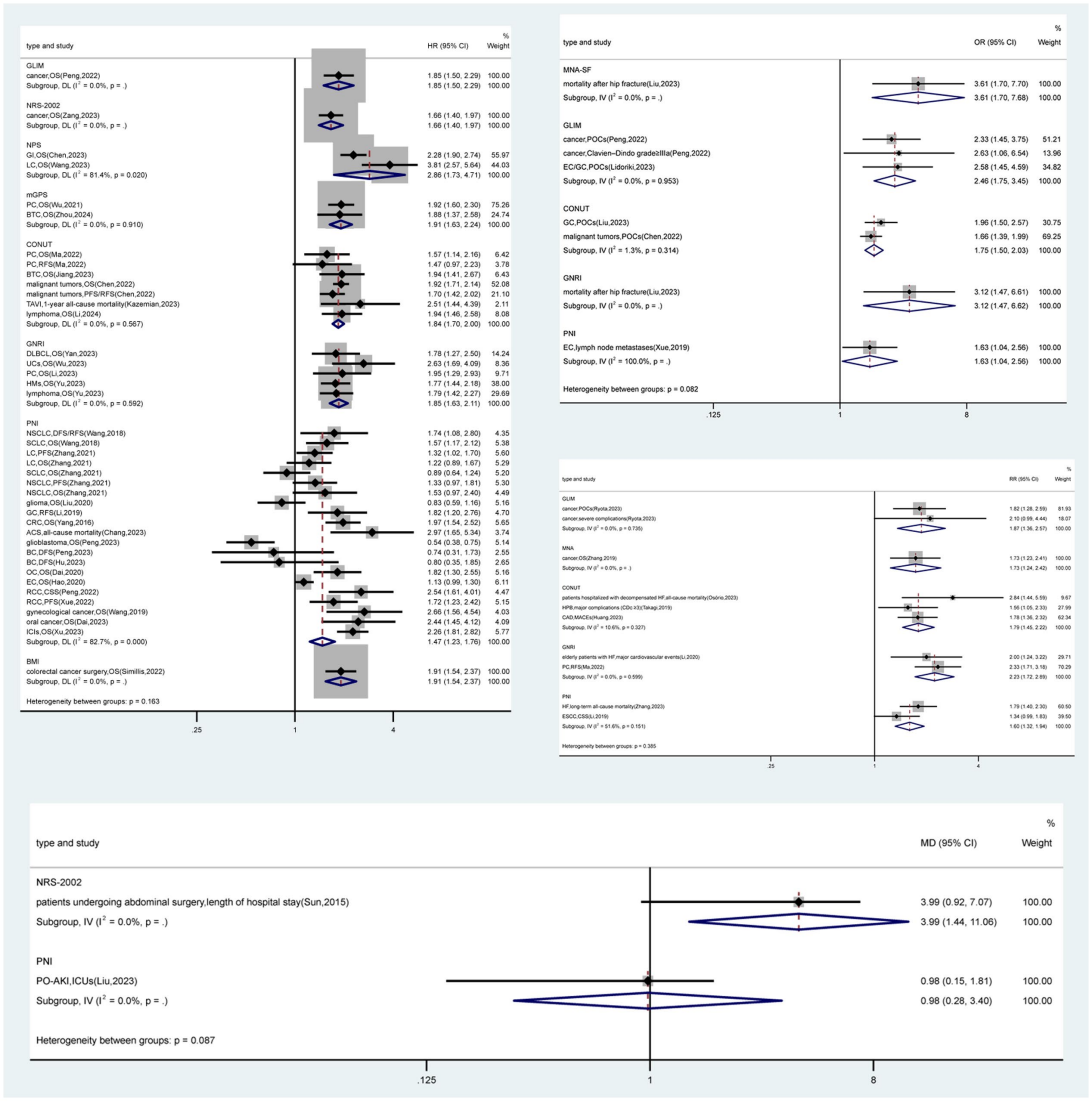
The forest plot shows the results of the suggestive evidence obtained from a comprehensive evaluation of nutritional screening assessment tools and clinical outcomes. Data are expressed as hazard/risk/odds ratios and 95% confidence intervals. BC, breast cancer; BTC, biliary tract carcinoma; ACS, acute coronary syndrome; CAD, coronary artery disease; CRC, colorectal cancer; CSS, cancer-specific survival; DFS, disease free survival; DLBCL, diffuse large B-cell lymphoma; EC, esophageal cancer; ESCC, esophageal squamous cell carcinoma; GC, gastric cancer; GI, gastrointestinal cancers; HF, heart failure; HMs, hematologic malignancies; HPB, hepatopancreatobiliary; ICIs, patients treated with immune checkpoint inhibitors; ICUS, intensive care unit stay; LC, lung cancer; MACEs, major adverse cardiovascular events; NSCLC, non-small cell lung cancer; OC, ovarian cancer; OS, overall survival; PC, pancreatic cancer; PFS, progression-free survival; PO-AKI, postoperative acute kidney injury; POCs, postoperative complications; RCC, renal cell carcinoma; RFS, relapse-free survival; SCLC, small cell lung cancer; TAVI, patients who underwent transcatheter aortic valve implantation; UCs, urological cancers.

Recommendations for diagnostic tools for malnutrition (Figures 3, 4): (1) In oncology and surgical patients, malnutrition diagnosed by the PNI, GNRI, and CONUT is strongly associated with survival (Class II) and is highly recommended (20, 88, 89, 92–98, 102, 103, 106, 107); malnutrition diagnosed by the GLIM, NRS 2002, NPS, mGPS, and MNA is associated with survival (Class III) and recommended (109, 111–116). (2) In nontumor patients, malnutrition diagnosed by the PNI, BMI < 18.5 kg/m^2^, GNRI, and CONUT is strongly associated with poor clinical outcomes in nontumor disease (Class II) and highly recommended (90, 91, 99, 101, 104, 105); malnutrition diagnosed by the PNI, GNRI, CONUT, MNA-SF, and NRS-2002 is associated with poor clinical outcomes in nontumor disease (Class III) and recommended (38, 100, 105, 108, 121, 122, 124, 127, 133, 147). (3) Under resource-constrained conditions, BMI/GNRI may be considered for assessment.

## 5 Conclusion

For oncology patients, malnutrition diagnosed by eight tools was associated with oncological survival, with three evidence scores of the PNI, GNRI, and CONUT being highly recommended (Class II), followed by five evidence scores of the GLIM, NRS 2002, NPS, mGPS, and MNA being recommended (Class III). For nontumor patients, malnutrition diagnosed by nine tools was associated with adverse clinical outcomes, with four tools (PNI, BMI < 18.5 kg/m^2^, GNRI, and CONUT) providing evidence of highly recommended (Class II), and five tools (PNI, GNRI, CONUT, MNA-SF, and NRS 2002) providing evidence of recommended (Class III). These tools are developed based on clinical presentation and signs of fluid and blood indicators, and screening for nutritional risk or risk of malnutrition, followed by malnutrition diagnosis, needs to be considered before selecting the tool, considering the predictability and accuracy of the tool, such as the GLIM. How to select a reasonable malnutrition diagnostic tool still requires relevant and high-quality evidence in a broader disease population to provide clinical experts with the ability to select a malnutrition diagnostic tool to provide favorable evidence.

## Data Availability

The original contributions presented in the study are included in the article/Supplementary material, further inquiries can be directed to the corresponding authors.

## Glossary

AMSTAR-2: a measurement tool to assess systematic reviews version 2
BMI: body mass index
CI: confidence intervals
CONUT: controlling nutritional status
CRP: C-reactive protein
ESS: effect size and significance
GLIM: global leadership initiative on malnutrition
GNRI: geriatric nutritional risk index
GPS: Glasgow Prognostic Score
GRADE: grading of recommendations assessment development and evaluation
MACEs: major adverse cardiovascular events
mGPS: modified Glasgow Prognostic Score
MNA: mini-nutritional assessment
MNA-SF: mini-nutritional assessment short-form
NOS: Newcastle–Ottawa Scale
NPS: Naples prognostic score
NRS 2002: nutritional risk screening 2002
PA: phase angle
PI: prediction intervals
PNI: prognostic nutritional index
PO-AKI: postoperative acute kidney injury
PSST: p-curve and statistical standards
ROBINS-I: risk of bias in non-randomized studies-of interventions
SE: standard error
SGA: subjective global assessment
SIGN: Scottish Intercollegiate Guidelines Network
TAVI: transcatheter aortic valve implantation
