# Supplementary material for: Association between malnutrition diagnosed by different screening and assessment tools and clinical outcomes: an umbrella review
Source: Front Nutr. 2025 Oct 9;12:1676201. doi: 10.3389/fnut.2025.1676201 (PMC12545068; doi:10.3389/fnut.2025.1676201)
Supplement: Supplementary file 1 [file Table_1.DOCX]

**Supplementary Figure1:** Search strings.

| Database | Search strings |
| --- | --- |
| Pubmed | (“Nutritional Risk[MeSH Terms]” OR “Nutritional Screening[MeSH Terms]” OR “(((Nutritional Status[MeSH Terms]) OR (Status, Nutritional[Title/Abstract])) OR (Nutrition Status[Title/Abstract])) OR (Status, Nutrition[Title/Abstract])” OR “(((((((((((((((((((((((((((((((((((((((((Nutrition Assessment[MeSH Terms]) OR (Assessments, Nutrition[Title/Abstract])) OR (Nutrition Assessments[Title/Abstract])) OR (Nutritional Assessment[Title/Abstract])) OR (Assessment, Nutritional[Title/Abstract])) OR (Assessments, Nutritional[Title/Abstract])) OR (Nutritional，Assessments[Title/Abstract])) OR (Assessment, Nutrition[Title/Abstract])) OR (Nutrition Indexes[Title/Abstract])) OR (Indexes, Nutrition[Title/Abstract])) OR (Nutrition Indices[Title/Abstract])) OR (Nutritional Index[Title/Abstract])) OR (Index, Nutritional[Title/Abstract])) OR (Indices, Nutritional[Title/Abstract])) OR (Nutritional Indices[Title/Abstract])) OR (Nutrition Index[Title/Abstract])) OR (Index, Nutrition[Title/Abstract])) OR (Indices, Nutrition[Title/Abstract])) OR (Prognostic Nutritional Index (PNI[Title/Abstract]))) OR (Index, Prognostic Nutritional (PNI[Title/Abstract]))) OR (Indices, Prognostic Nutritional (PNI[Title/Abstract]))) OR (Nutritional Index, Prognostic (PNI[Title/Abstract]))) OR (Nutritional Indices, Prognostic (PNI[Title/Abstract]))) OR (Prognostic Nutritional Indices (PNI[Title/Abstract]))) OR (Prognostic Nutritional Index[Title/Abstract])) OR (Index, Prognostic Nutritional[Title/Abstract])) OR (Indices, Prognostic Nutritional[Title/Abstract])) OR (Nutritional Index, Prognostic[Title/Abstract])) OR (Nutritional Indices, Prognostic[Title/Abstract])) OR (Prognostic Nutritional Indices[Title/Abstract])) OR (Mini Nutritional Assessment[Title/Abstract])) OR (Assessment, Mini Nutritional[Title/Abstract])) OR (Assessments, Mini Nutritional[Title/Abstract])) OR (Mini Nutritional Assessments[Title/Abstract])) OR (Nutritional Assessment, Mini[Title/Abstract])) OR (Nutritional Assessments, Mini[Title/Abstract])) OR (Mini Nutrition Assessment[Title/Abstract])) OR (Assessment, Mini Nutrition[Title/Abstract])) OR (Assessments, Mini Nutrition[Title/Abstract])) OR (Mini Nutrition Assessments[Title/Abstract])) OR (Nutrition Assessment, Mini[Title/Abstract])) OR (Nutrition Assessments, Mini[Title/Abstract])” OR “(((((Malnutrition[MeSH Terms]) OR (Nutritional Deficiency[Title/Abstract])) OR (Nutritional Deficiencies[Title/Abstract])) OR (Undernutrition[Title/Abstract])) OR (Malnourishment[Title/Abstract])) OR (Malnourishments[Title/Abstract])” and “meta-analysis” OR “systematic review”)) |
| Embase | (‘Nutritional Risk’/ OR ‘Nutritional Screening’/ OR ‘Nutritional Status’/ OR ‘Nutritional Status’:ab,kw,ti OR ‘Status, Nutritional’:ab,kw,ti OR ‘Nutrition Status’:ab,kw,ti OR ‘Status, Nutrition’:ab,kw,ti OR Nutrition Assessment/ OR ‘Nutrition Assessment’:ab,kw,ti OR ‘Assessments, Nutrition’:ab,kw,ti OR ‘Nutrition Assessments’:ab,kw,ti OR ‘Nutritional Assessment’:ab,kw,ti OR ‘Assessment, Nutritional’:ab,kw,ti OR ‘Assessments, Nutritional’:ab,kw,ti OR ‘Nutritional, Assessments’:ab,kw,ti OR ‘Assessment, Nutrition’:ab,kw,ti OR ‘Nutrition Indexes’:ab,kw,ti OR ‘Indexes, Nutrition’:ab,kw,ti OR ‘Nutrition Indices’:ab,kw,ti OR ‘Nutritional Index’:ab,kw,ti OR ‘Index, Nutritional’:ab,kw,ti OR ‘Indices, Nutritional’:ab,kw,ti OR ‘Nutritional Indices’:ab,kw,ti OR ‘Nutrition Index’:ab,kw,ti OR ‘Index, Nutrition’:ab,kw,ti OR ‘Indices, Nutrition’:ab,kw,ti OR ‘Prognostic Nutritional Index (PNI)’:ab,kw,ti OR ‘Index, Prognostic Nutritional (PNI)’:ab,kw,ti OR ‘Indices, Prognostic Nutritional (PNI)’:ab,kw,ti OR ‘Nutritional Index, Prognostic (PNI)’:ab,kw,ti OR ‘Nutritional Indices, Prognostic (PNI)’:ab,kw,ti OR ‘Prognostic Nutritional Indices (PNI)’:ab,kw,ti OR ‘Prognostic Nutritional Index’:ab,kw,ti OR ‘Index, Prognostic Nutritional’:ab,kw,ti OR ‘Indices, Prognostic Nutritional’:ab,kw,ti OR ‘Nutritional Index, Prognostic’:ab,kw,ti OR ‘Nutritional Indices, Prognostic’:ab,kw,ti OR ‘Prognostic Nutritional Indices’:ab,kw,ti OR ‘Mini Nutritional Assessment’:ab,kw,ti OR ‘Assessment, Mini Nutritional’:ab,kw,ti OR ‘Assessments, Mini Nutritional’:ab,kw,ti OR ‘Mini Nutritional Assessments’:ab,kw,ti OR ‘Nutritional Assessment, Mini’:ab,kw,ti OR ‘Nutritional Assessments, Mini’:ab,kw,ti OR ‘Mini Nutrition Assessment’:ab,kw,ti OR ‘Assessment, Mini Nutrition’:ab,kw,ti OR ‘Assessments, Mini Nutrition’:ab,kw,ti OR ‘Mini Nutrition Assessments’:ab,kw,ti OR ‘Nutrition Assessment, Mini’:ab,kw,ti OR ‘Nutrition Assessments, Mini’:ab,kw,ti OR Malnutrition/ OR‘Malnutrition’:ab,kw,ti OR ‘Nutritional Deficiency’:ab,kw,ti OR ‘Nutritional Deficiencies’:ab,kw,ti OR ‘Undernutrition’:ab,kw,ti OR ‘Malnourishment’:ab,kw,ti OR ‘Malnourishments’) AND (‘meta-analysis’/ OR ‘systematic review’/)) |
| Cochrane | (“Nutritional Risk[MeSH Terms]” OR “Nutritional Screening[MeSH Terms]” OR “(((Nutritional Status[MeSH Terms]) OR (Status, Nutritional[Title/Abstract])) OR (Nutrition Status[Title/Abstract])) OR (Status, Nutrition[Title/Abstract])” OR “(((((((((((((((((((((((((((((((((((((((((Nutrition Assessment[MeSH Terms]) OR (Assessments, Nutrition[Title/Abstract])) OR (Nutrition Assessments[Title/Abstract])) OR (Nutritional Assessment[Title/Abstract])) OR (Assessment, Nutritional[Title/Abstract])) OR (Assessments, Nutritional[Title/Abstract])) OR (Nutritional，Assessments[Title/Abstract])) OR (Assessment, Nutrition[Title/Abstract])) OR (Nutrition Indexes[Title/Abstract])) OR (Indexes, Nutrition[Title/Abstract])) OR (Nutrition Indices[Title/Abstract])) OR (Nutritional Index[Title/Abstract])) OR (Index, Nutritional[Title/Abstract])) OR (Indices, Nutritional[Title/Abstract])) OR (Nutritional Indices[Title/Abstract])) OR (Nutrition Index[Title/Abstract])) OR (Index, Nutrition[Title/Abstract])) OR (Indices, Nutrition[Title/Abstract])) OR (Prognostic Nutritional Index (PNI[Title/Abstract]))) OR (Index, Prognostic Nutritional (PNI[Title/Abstract]))) OR (Indices, Prognostic Nutritional (PNI[Title/Abstract]))) OR (Nutritional Index, Prognostic (PNI[Title/Abstract]))) OR (Nutritional Indices, Prognostic (PNI[Title/Abstract]))) OR (Prognostic Nutritional Indices (PNI[Title/Abstract]))) OR (Prognostic Nutritional Index[Title/Abstract])) OR (Index, Prognostic Nutritional[Title/Abstract])) OR (Indices, Prognostic Nutritional[Title/Abstract])) OR (Nutritional Index, Prognostic[Title/Abstract])) OR (Nutritional Indices, Prognostic[Title/Abstract])) OR (Prognostic Nutritional Indices[Title/Abstract])) OR (Mini Nutritional Assessment[Title/Abstract])) OR (Assessment, Mini Nutritional[Title/Abstract])) OR (Assessments, Mini Nutritional[Title/Abstract])) OR (Mini Nutritional Assessments[Title/Abstract])) OR (Nutritional Assessment, Mini[Title/Abstract])) OR (Nutritional Assessments, Mini[Title/Abstract])) OR (Mini Nutrition Assessment[Title/Abstract])) OR (Assessment, Mini Nutrition[Title/Abstract])) OR (Assessments, Mini Nutrition[Title/Abstract])) OR (Mini Nutrition Assessments[Title/Abstract])) OR (Nutrition Assessment, Mini[Title/Abstract])) OR (Nutrition Assessments, Mini[Title/Abstract])” OR “(((((Malnutrition[MeSH Terms]) OR (Nutritional Deficiency[Title/Abstract])) OR (Nutritional Deficiencies[Title/Abstract])) OR (Undernutrition[Title/Abstract])) OR (Malnourishment[Title/Abstract])) OR (Malnourishments[Title/Abstract])” and “meta-analysis” OR “systematic review”)) |
| Sciencedirect | ("Nutritional Risk" OR "Nutritional Screening" OR "Nutritional Status" OR "Nutrition Assessment" OR "Malnutrition") AND ("meta-analysis" OR "systematic review") |
| Web of science | (TS= (Nutritional Risk)) OR (TS=(Nutritional Screening)) OR (TS=(Nutritional Status) OR AB=(Status, Nutritional OR Nutrition Status OR Status, Nutrition)) OR (TS=(Nutrition Assessment) OR AB=(Assessments, Nutrition OR Nutrition Assessments OR Nutritional Assessment OR Assessment, Nutritional OR Assessments, Nutritional OR Nutritional，Assessments OR Assessment, Nutrition OR Nutrition Indexes OR Indexes, Nutrition OR Nutrition Indices OR Nutritional Index OR Index, Nutritional OR Indices, Nutritional OR Nutritional Indices OR Nutrition Index OR Index, Nutrition OR Indices, Nutrition OR Prognostic Nutritional Index (PNI) OR Index, Prognostic Nutritional (PNI) OR Indices, Prognostic Nutritional (PNI) OR Nutritional Index, Prognostic (PNI) OR Nutritional Indices, Prognostic (PNI) OR Prognostic Nutritional Indices (PNI) OR Prognostic Nutritional Index OR Index, Prognostic Nutritional OR Indices, Prognostic Nutritional OR Nutritional Index, Prognostic OR Nutritional Indices, Prognostic OR Prognostic Nutritional Indices OR Mini Nutritional Assessment OR Assessment, Mini Nutritional OR Assessments, Mini Nutritional OR Mini Nutritional Assessments OR Nutritional Assessment, Mini OR Nutritional Assessments, Mini OR Mini Nutrition Assessment OR Assessment, Mini Nutrition OR Assessments, Mini Nutrition OR Mini Nutrition Assessments OR Nutrition Assessment, Mini OR Nutrition Assessments, Mini)) OR (TS=(Malnutrition) OR AB=(Nutritional Deficiency OR Nutritional Deficiencies OR Undernutrition OR Malnourishment OR Malnourishments)) AND (TI=(meta-analysis OR systematic review)) |
| CNKI | ("Nutritional Risk" OR "Nutritional Screening" OR "Nutritional Status" OR "Nutrition Assessment" OR "Malnutrition") AND ("meta-analysis" OR "systematic review") |
| VIP database | ("Nutritional Risk" OR "Nutritional Screening" OR "Nutritional Status" OR "Nutrition Assessment" OR "Malnutrition") AND ("meta-analysis" OR "systematic review") |
