# Supplementary material for: Association between malnutrition diagnosed by different screening and assessment tools and clinical outcomes: an umbrella review
Source: Front Nutr. 2025 Oct 9;12:1676201. doi: 10.3389/fnut.2025.1676201 (PMC12545068; doi:10.3389/fnut.2025.1676201)
Supplement: Supplementary file 2 [file Table_2.DOCX]

**Supplementary Figure2:** Characteristics of included studies.

| First author | Year | Research type | No. of studies | No. of cases | | Age, year ± SD | Male (%) | Quality Assessment |
| --- | --- | --- | --- | --- | --- | --- | --- | --- |
| **A] Mini-nutritional assessment short-form** **(MNA-SF)** | | | | |  |  |  |  |
| Liu(1) | 2023 | observational studies | 13 | 4481 | | 77.6~87.5 | 18.7~43.8 | NOS |
| Hu(2) | 2022 | Cohort studies | 31 | 25141 | | 18 | 43.7~89.2 | NOS |
| Osório(3) | 2023 | prospective and retrospective cohort studies | 38 | 30043 | | 39.4~78.1 | 41~92.1 | NOS |
| Lin(4) | 2016 | cohort or cross-sectional studies | 11 | 4300 | | U | 24~80 | NOS |
| **B] Subjective global assessment** **(SGA)** | | | | |  |  |  |  |
| Hu(2) | 2022 | Cohort studies | 31 | 25141 | | 18 | 43.7~89.2 | NOS |
| **C] The Global Leadership Initiative on Malnutrition (GLIM)** | | | | | | | | |
| Peng(5) | 2022 | retrospective or prospective observational studies | 15 | 14573 | | 52.79~73.67 | U | NOS |
| Ryota(6) | 2023 | prospective or retrospective cohort and case-control studies | 12 | 11700 | | 56.9~70.1 | 26~75.2 | GRADE |
| Xu(7) | 2022 | cohort studies | 12 | 6799 | | 39.5~81.8 | 26~75.2 | ROBINS-I |
| Yin(8) | 2023 | retrospective and prospective cohort studies | 9 | 8829 | | 56.9~66 | U | NOS |
| Lidoriki(9) | 2023 | Prospective and retrospective observational studies | 7 | 3662 | | 47.4~76 | U | NOS |
| **D] Nutritional risk screening (****NRS 2002)** | | | | |  |  |  |  |
| Sun(10) | 2015 | Prospective or retrospective cohort studies | 11 | 3527 | | 51~66 | 53~74 | NOS |
| Zang(11) | 2023 | cohort studies or post hoc analysis of randomized controlled trials | 22 | 9332 | | 48.5~67.6 | 0~94.7 | NOS |
| **E] The naples prognostic score (****NPS)** | | | | |  |  |  |  |
| Chen(12) | 2023 | U | 13 | 7321 | | >57.8 | 54.4~87.1 | NOS |
| Wang(13) | 2023 | U | 7 | 1657 | | 57~65 | 44.8~75.8 | NOS |
| **F] modified Glasgow Prognostic Score (****mGPS)** | | | | |  |  |  |  |
| Wu(14) | 2021 | prospective and retrospective studies | 25 | 4629 | | U | U | NOS |
| Nie(15) | 2020 | prospective and retrospective studies | 11 | 2830 | | 16-93 | U | NOS |
| Hu(16) | 2019 | U | 12 | 2391 | | 24-89 | U | NOS |
| Zhou(17) | 2024 | retrospective or prospective studies | 15 | 2447 | |  |  |  |
| **G] Mini-nutritional assessment（****MNA）** | | | | |  |  |  |  |
| Zhang(18) | 2019 | retrospective and prospective cohort studies | 10 | 4692 | | 75~80 | U | NOS |
| Hu(2) | 2022 | Cohort studies | 31 | 25141 | | 18 | 43.7~89.2 | NOS |
| Lin(4) | 2016 | cohort or cross-sectional studies | 11 | 4300 | | U | 24~80 | NOS |
| **H] Controlling nutritional status (****CONUT) score** | | | | |  |  |  |  |
| Ma(19) | 2022 | U | 7 | 2294 | | 28~85 | U | NOS |
| Niu(20) | 2021 | U | 12 | 6405 | | U | U | NOS |
| Liu(21) | 2023 | retrospective single center and multiple center | 19 | 9764 | | 21~94 | 64.9~82.4 | U |
| Yin(22) | 2023 | U | 17 | 10009 | | U | U | NOS |
| Takagi(23) | 2019 | Retrospective  Single center | 5 | 2482 | | U | 64.2~75.8 | NOS |
| Liu(24) | 2023 | Retrospective | 9 | 1409 | | U | U | NOS |
| Jiang(25) | 2023 | U | 10 | 1441 | | 23~95 | U | NOS |
| Chen(26) | 2022 | U | 62 | 25224 | | U | U | NOS |
| Chen(27) | 2022 | U | 6 | 3529 | | U | 45.4~71.2 | NOS |
| Xue(28) | 2022 | U | 7 | 5410 | | 42~85 | 45.4~72.4 | Cochrane Handbook |
| peng(29) | 2022 | retrospective study or prospective study or randomized controlled study | 7 | 5410 | | 42~85 | U | NOS |
| Shao(30) | 2021 | U | 8 | 1220 | | U | U | NOS |
| Zhang(31) | 2021 | retrospective single center | 8 | 1836 | | 29~93 | 56.6~90.6 | NOS |
| Takagi(32) | 2020 | retrospective single center | 6 | 3608 | | U | 54.9~59.7 | NOS |
| TAKAGI(33) | 2020 | retrospective single center | 5 | 1304 | | U | 48.0~89.2 | NOS |
| Lv(34) | 2023 | U | 11 | 3783 | | U | U | N0S |
| Niu(35) | 2023 | retrospective or prospective studies | 6 | 2569 | | 32~67 | U | NOS |
| Lu(36) | 2022 | retrospective single center | 6 | 1811 | | 20~97 | U | NOS |
| Peng(37) | 2021 | retrospective | 11 | 3029 | | U | U | NOS |
| Li(38) | 2022 | prospective or retrospective observational studies | 10 | 5196 | | 61~80 | U | NOS |
| Hu(2) | 2022 | Cohort studies | 31 | 25141 | | 18 | 43.7~89.2 | NOS |
| Peng(39) | 2023 | U | 10 | 1406 | | 49.9~63 | U | NOS |
| Osório(3) | 2023 | prospective and retrospective cohort studies | 38 | 30043 | | 39.4~78.1 | 41~92.1 | NOS |
| Kazemian(40) | 2023 | observational studies | 13 | 6785 | | 69.3~91.2 | 29.5~59.6 | NOS |
| Ni(41) | 2022 | cohort studies | 26 | 17647 | | 52.8~90.71 | 22.4~76.5 | NOS |
| Peng(42) | 2023 | cohort studies and cross-sectional studies and case-control studies | 8 | 5301 | | 47~66 | U | NOS |
| Takagi(43) | 2019 | retrospective single center | 10 | 5138 | | U | 54.9~89.2 | NOS |
| Zhang(44) | 2018 | U | 9 | 2400 | | 64~68 | 54.9~89.2 | NOS |
| Li(45) | 2024 | U | 7 | 2060 | | 16~97 | 50.8~84.6 | NOS |
| Jiao(46) | 2023 | U | 13 | 4413 | | 60.7~82 | 67.7~87.5 | NOS |
| Huang(47) | 2023 | retrospective or prospective observational studies | 12 | 36198 | | 51~83.6 | 51.6~88.1 | NOS |
| **I] Geriatric nutritional risk index (****GNRI)** | | | | |  |  |  |  |
| Yan(48) | 2023 | cohort studies | 7 | 2448 | | 44.7~78 | 49.4~55.9 | NOS |
| Cao(49) | 2023 | U | 7 | 2353 | | 20~97 | 49.4~58.4 | NOS |
| Shen(50) | 2022 | U | 11 | 2865 | | 65~71.2 | 23.7~87 | NOS |
| Yang(51) | 2022 | prospective and retrospective cohort studies | 11 | 2865 | | 62.6~78 | 43.3~85.6 | NOS |
| Yiu(52) | 2023 | U | 10 | 2793 | | 28~85 | 61.3~87.2 | NOS |
| Mao(53) | 2022 | U | 10 | 3802 | | 62.4~83 | 43.4~79.6 | NOS |
| Yuan(54) | 2023 | U | 9 | 3440 | | 30~94 | U | NOS |
| Xu(55) | 2023 | prospective or retrospective cohort studies | 8 | 3239 | | 32~94 | 52.5~73 | NOS |
| Zhao(56) | 2022 | prospective and retrospective cohort studies | 9 | 3658 | | 63~80.5 | 45~73 | NOS |
| Wu(57) | 2023 | retrospective study | 8 | 6792 | | 27~87 | 64.1~82.3 | NOS |
| He(58) | 2023 | U | 10 | 5834 | | 35~95 | 63.4~78.3 | NOS |
| Lu(59) | 2022 | U | 8 | 4189 | | 28~91 | U | NOS |
| Zhang(60) | 2023 | retrospective study | 11 | 5593 | | 25~96 | 63.4~72.9 | NOS |
| Wang(61) | 2022 | U | 8 | 2399 | | U | U | NOS |
| Wang(62) | 2022 | U | 8 | 2012 | | U | U | NOS |
| Xie(63) | 2020 | U | 9 | 2153 | | 30~94 | U | NOS |
| Fan(64) | 2021 | cohort studies | 8 | 9277 | | 45.5~81 | 63.1~83.6 | NOS |
| Liu(65) | 2022 | retrospective or prospective observational studies | 8 | 5541 | | 61~89.2 | 47~80.4 | NOS |
| Zhou(66) | 2022 | observational studies or randomized controlled trials (RCTs) | 8 | 1785 | | 55.6~83 | 61.6~88.5 | NOS |
| Yang(67) | 2022 | U | 8 | 1354 | | U | 62.8~88.5 | NOS |
| Yu(68) | 2022 | U | 14 | 3981 | | ≥60 | U | NOS |
| Fan(69) | 2022 | longitudinal observational studies | 8 | 1460 | | 47~89 | 62.8~88.5 | NOS |
| Li(70) | 2021 | prospective or retrospective observational study | 11 | 10589 | | 53~92.1 | 28.7~77 | NOS |
| Osório(3) | 2023 | prospective and retrospective cohort studies | 38 | 30043 | | 39.4~78.1 | 41~92.1 | NOS |
| LIU(1) | 2023 | observational studies | 13 | 4481 | | 77.6~87.5 | 18.7~43.8 | NOS |
| Hu(2) | 2022 | Cohort studies | 31 | 25141 | | 18 | 43.7~89.2 | NOS |
| Lv(71) | 2019 | U | 15 | 8046 | | 18~113 | U | U |
| Yuan(72) | 2021 | Cohort studies | 20 | 10162 | | 40.8~79.8 | 50.1~71.7 | NOS |
| Kazemian(40) | 2023 | observational studies | 13 | 6785 | | 69.3~91.2 | 29.5~59.6 | NOS |
| Ni(41) | 2022 | cohort studies | 26 | 17647 | | 52.8~90.71 | 22.4~76.5 | NOS |
| Lin(4) | 2016 | cohort or cross-sectional studies | 11 | 4300 | | U | 24~80 | NOS |
| Li(73) | 2023 | Prospective and retrospective studies | 6 | 1513 | | 34~89 | 41.5~57.8 | NOS |
| Yu(74) | 2023 | Prospective cohort study or randomized controlled trial or retrospective study | 14 | 3524 | | 19~97 | 46.2~70.9 | NOS |
| Liu(75) | 2024 | Cohort or case-control studies | 11 | 11002 | | 54.8~94 | 31.1~100 | NOS |
| **J] Prognostic Nutritional Index (****PNI)** | | | | |  |  |  |  |
| Luan(76) | 2020 | U | 7 | 1311 | | 16~94 | U | NOS |
| Jiang(77) | 2020 | case-control or cohort studies | 9 | 4164 | | 16~86 | 64.4~89.9 | NOS |
| Ren(78) | 2023 | retrospective studies | 8 | 2462 | | 18~94 | U | NOS |
| Kang(79) | 2022 | U | 8 | 2307 | | 18~94 | 42.2~81.7 | NOS |
| Li(80) | 2022 | retrospective studies | 8 | 2627 | | 19~94 | 44.6~57.6 | NOS |
| Hu(81) | 2018 | U | 15 | 4736 | | 57~79 | U | NOS |
| Wang(82) | 2018 | retrospective studies | 21 | 7408 | | 56~70.5 | 59.0~86.6 | NOS |
| Zhang(83) | 2021 | Case-control studies or prospective cohort trials or retrospective cohort trials | 16 | 4250 | | 46.2~79.2 | 66.3 | NOS |
| Wang(84) | 2020 | U | 18 | 3261 | | 41.6~62.9 | 45.2~80.6 | NOS |
| Liu(85) | 2020 | U | 11 | 2928 | | 21~91 | 50.6~63.1 | NOS |
| Hung(86) | 2023 | Observational studies | 13 | 2712 | | 18~82 | 50.5~64.5 | NOS |
| Li(87) | 2019 | prospective and retrospective cohort studies | 25 | 14403 | | 14~96 | U | NOS |
| Yang(88) | 2016 | U | 10 | 3396 | | 19~89 | 64.0~76.8 | NOS |
| Lv(89) | 2021 | retrospective studies | 7 | 1608 | | 29~87 | 32.1~63 | NOS |
| Qi(90) | 2018 | cohort studies or case–control studies | 12 | 6561 | | U | U | NOS |
| Sun(91) | 2019 | comparative studies (retrospective and prospective studies) | 10 | 6372 | | U | 47~78 | NOS |
| Yang(92) | 2016 | cohort studies | 11 | 3788 | | 50.2~71.2 | 55.8~61.5 | NOS |
| Li(93) | 2019 | retrospective studies | 10 | 2064 | | U | U | NOS |
| Liu(1) | 2023 | observational studies | 13 | 4481 | | 77.6~87.5 | 18.7~43.8 | NOS |
| Hu(2) | 2022 | Cohort studies | 31 | 25141 | | 18 | 43.7~89.2 | NOS |
| Ni(41) | 2022 | cohort studies | 26 | 17647 | | 52.8~90.71 | 22.4~76.5 | NOS |
| Zhang(94) | 2023 | retrospective or prospective observational study | 12 | 9365 | | 58~90.75 | 39.4~68 | NOS |
| Chen(95) | 2022 | observational studies | 14 | 19605 | | 60.9~84 | U | NOS |
| Osório(3) | 2023 | prospective and retrospective cohort studies | 38 | 30043 | | 39.4~78.1 | 41~92.1 | NOS |
| Chang(96) | 2023 | Retrospective observational studies | 13 | 16579 | | 58~73.1 | 51.6~81 | NOS |
| Shi(97) | 2021 | cohort studies | 10 | 3458 | | 44~79 | 48.9~96.7 | NOS |
| Luan(98) | 2021 | U | 14 | 5891 | | U | U | NOS |
| Peng(39) | 2023 | U | 10 | 1406 | | 49.9~63 | U | NOS |
| Kazemian(40) | 2023 | observational studies | 13 | 6785 | | 69.3~91.2 | 29.5~59.6 | NOS |
| Peng(42) | 2023 | cohort studies and cross-sectional studies and case-control studies | 8 | 5301 | | 47~66 | U | NOS |
| Hu(99) | 2023 | U | 8 | 2322 | | 9.2~96 | 0 | NOS |
| Meng(100) | 2022 | retrospective or prospective studies | 6 | 2324 | | 29.5`90 | U | NOS |
| Dai(101) | 2020 | retrospective or prospective studies | 6 | 2050 | | 50~56 | U | NOS |
| Tan(102) | 2023 | retrospective and prospective observational studies | 12 | 3190 | | U | U | NOS |
| Fan(103) | 2021 | Case-control studies | 7 | 187 | | 29~85 | U | NOS |
| Man(104) | 2018 | observational study | 13 | 3738 | | U | U | NOS |
| Liao(105) | 2020 | Observation study or prospective study or randomized controlled trials | 12 | 3118 | | 54.7~66.5 | U | NOS |
| Hao(106) | 2020 | U | 13 | 3543 | | 61.8~67.1 | 69.5~91.7 | NOS |
| Xue(107) | 2019 | U | 11 | 3425 | | 59~65.9 | U | NOS |
| Li(108) | 2019 | U | 9 | 2276 | | 33~92 | 76.1`90.1 | NOS |
| Mao(109) | 2021 | U | 10 | 4908 | | 14~87 | U | NOS |
| Kim(110) | 2021 | clinical trial and prospective or retrospective study | 9 | 5876 | | 14~87 | 59.8~79.2 | NOS |
| Xiong(111) | 2022 | retrospective studies | 12 | 7391 | | 14~88 | U | NOS |
| Peng(112) | 2022 | retrospective studies | 11 | 7629 | | U | 38.8~79.2 | NOS |
| Xue(113) | 2022 |  | 16 | 11781 | | U | U | NOS |
| Wang(114) | 2019 | Prospective and observational retrospective studies | 9 | 2373 | | 22~90 | U | NOS |
| Zheng(115) | 2023 | retrospective studies | 10 | 1631 | | 46~86 | U | NOS |
| Dai(116) | 2023 | retrospective studies | 10 | 3130 | | 20~93 | 48.9~90.3 | NOS |
| Tang(117) | 2021 | U | 8 | 3631 | | 12~82 | U | NOS |
| Tu(118) | 2020 | U | 10 | 4511 | | U | U | NOS |
| Gao(119) | 2021 | retrospective cohort studies | 8 | 4299 | | 13~82 | 51.1~83.4 | NOS |
| Zhang(120) | 2023 | U | 15 | 22521 | | 58~73.1 | 51.6~82.9 | NOS |
| Sun(121) | 2014 | U | 14 | 3414 | | U | U | U |
| Bullock(122) | 2020 | observational studies | 42 | 21032 | | U | 46 | the Critical Appraisal Skills Program checklist items 1–10 |
| Guo(123) | 2024 | prospective and retrospective cohort studies | 10 | 3249 | | 53.9~78.9 | 16.4~71.8 | NOS |
| Jiao(46) | 2023 | U | 13 | 4413 | | 60.7~82 | 67.7~87.5 | NOS |
| Zhao(124) | 2022 | retrospective studies | 14 | 3385 | | U | U | NOS |
| Liu(125) | 2023 | Randomized controlled studies or observational cohort studies | 13 | 9185 | | 44~69 | 56.8~82.9 | NOS |
| Li(126) | 2022 | retrospective observational studies | 9 | 640 | | U | 65.5~93.3 | NOS |
| Ni(127) | 2022 | cohort studies | 12 | 1359 | | 59.9~73 | U | NOS |
| Yan(128) | 2023 | case-control or cohort studies | 14 | 1260 | | 59.9 - 75 | U | NOS |
| Xu(129) | 2023 | retrospective, prospective cohort, or randomized controlled trial | 23 | 2386 | | 52~73 | 49.8~93.3 | NOS |
| Zhang(130) | 2023 | observational studies | 17 | 2883 | | 15-94 | 54.3~93.3 | NOS |
| **K] Phase angle (****PA)** | | | | |  |  |  |  |
| Zheng(131) | 2023 | prospective and retrospective and cross-section studies | 20 | 3770 | | 43~70 | 48~82 | NOS |
| Arab(132) | 2021 | original human cohort studies | 11 | 2625 | | 50.2~70.9 | 40.9~86.3 | NOS |
| Fernández(133) | 2023 | U | 4 | U | | U | U | GRADE |
| **L] Body mass index (****BMI)** | | | | |  |  |  |  |
| Ahmadi(134) | 2016 | case-control or cohort or clinical trial studies | 61 | 156559 | | 18~94 | 46~57 | NOS |
| De Paola(135) | 2022 | prospective cohort studies | 24 | 308430 | | U | 30.6-83.8 | SIGN |
| Cao(136) | 2012 | cohort studies | 22 | 21150 | | 31~87 | U | NOS |
| Simillis(137) | 2022 | Prospective or retrospective comparative studies | 56 | 72582 | | U | U | NOS |
| He(138) | 2024 | prospective or retrospective cohort studies | 34 | 18343 | | 39~84 | 69.2~95.2 | NOS |

**Reference:**

1. Liu N, Lv L, Jiao J, Zhang Y, Zuo XL. Association between nutritional indices and mortality after hip fracture: a systematic review and meta-analysis. Eur Rev Med Pharmacol Sci. 2023;27(6):2297-304.

2. Hu Y, Yang H, Zhou Y, Liu X, Zou C, Ji S, et al. Prediction of all-cause mortality with malnutrition assessed by nutritional screening and assessment tools in patients with heart failure：a systematic review. Nutr Metab Cardiovasc Dis. 2022;32(6):1361-74.

3. Osório AF, Ribeiro ÉCT, Parahiba SM, Forte GC, Clausell NO, Souza GC. Prognostic value of nutritional screening tools in hospitalized patients with decompensated heart failure: A systematic review and meta-analysis. Nutr Res. 2023;120:1-19.

4. Lin H, Zhang H, Lin Z, Li X, Kong X, Sun G. Review of nutritional screening and assessment tools and clinical outcomes in heart failure. Heart Fail Rev. 2016;21(5):549-65.

5. Peng DD, Zong KZ, Yang H, Huang ZT, Mou T, Jiang P, et al. Malnutrition diagnosed by the Global Leadership Initiative on Malnutrition criteria predicting survival and clinical outcomes of patients with cancer: A systematic review and meta-analysis. Front Nutr. 2022;9:1053165.

6. Matsui R, Rifu K, Watanabe J, Inaki N, Fukunaga T. Impact of malnutrition as defined by the GLIM criteria on treatment outcomes in patients with cancer: A systematic review and meta-analysis. Clin Nutr. 2023;42(5):615-24.

7. Xu J, Jie Y, Sun Y, Gong D, Fan Y. Association of Global Leadership Initiative on Malnutrition with survival outcomes in patients with cancer: A systematic review and meta-analysis. Clin Nutr. 2022;41(9):1874-80.

8. Yin L, Chong F, Huo Z, Li N, Liu J, Xu H. GLIM-defined malnutrition and overall survival in cancer patients: A meta-analysis. JPEN J Parenter Enteral Nutr. 2023;47(2):207-19.

9. Lidoriki I, Frountzas M, Mela E, Papaconstantinou D, Vailas M, Sotiropoulou M, et al. The Prognostic Role of GLIM Criteria in Postoperative Outcomes after Upper Gastrointestinal Cancer Surgery: A Meta-Analysis of Observational Studies. Nutr Cancer. 2023;75(2):640-51.

10. Sun Z, Kong XJ, Jing X, Deng RJ, Tian ZB. Nutritional Risk Screening 2002 as a Predictor of Postoperative Outcomes in Patients Undergoing Abdominal Surgery: A Systematic Review and Meta-Analysis of Prospective Cohort Studies. PLoS One. 2015;10(7):e0132857.

11. Zang Y, Xu W, Qiu Y, Gong D, Fan Y. Association between Risk of Malnutrition Defined by the Nutritional Risk Screening 2002 and Postoperative Complications and Overall Survival in Patients with Cancer: A Meta-Analysis. Nutr Cancer. 2023;75(8):1600-9.

12. Chen FM, Xie C, Ren K, Xu XM. Prognostic Value of the Naples Prognostic Score in Patients with Gastrointestinal Cancers: A Meta-Analysis. Nutr Cancer. 2023;75(7):1520-30.

13. Wang YS, Niu L, Shi WX, Li XY, Shen L. Naples prognostic score as a predictor of outcomes in lung cancer: a systematic review and meta-analysis. Eur Rev Med Pharmacol Sci. 2023;27(17):8144-53.

14. Wu D, Wang X, Shi G, Sun H, Ge G. Prognostic and clinical significance of modified glasgow prognostic score in pancreatic cancer: a meta-analysis of 4,629 patients. Aging (Albany NY). 2021;13(1):1410-21.

15. Nie D, Zhang LP, Wang CY, Guo Q, Mao XG. A high Glasgow prognostic score (GPS) or modified Glasgow prognostic score (mGPS) predicts poor prognosis in gynecologic cancers: a systematic review and meta-analysis. Arch Gynecol Obstet. 2020;301(6):1543-51.

16. Hu X, Wang Y, Yang WX, Dou WC, Shao YX, Li X. Modified Glasgow prognostic score as a prognostic factor for renal cell carcinomas: a systematic review and meta-analysis. Cancer Manag Res. 2019;11(4):6163-73.

17. Zhou Y, Liu Z, Cheng Y, Li J, Fu W. Prognostic value of the modified Glasgow prognostic score in biliary tract cancer patients: a systematic review and meta-analysis. J Gastrointest Surg. 2024;28(4):559-65.

18. Zhang X, Tang T, Pang L, Sharma SV, Li R, Nyitray AG, et al. Malnutrition and overall survival in older adults with cancer: A systematic review and meta-analysis. J Geriatr Oncol. 2019;10(6):874-83.

19. Ma XF, Zou WH, Sun Y. Prognostic Value of Pretreatment Controlling Nutritional Status Score for Patients With Pancreatic Cancer: A Meta-Analysis. Front Oncol. 2022;11:770894.

20. Niu XH, Zhu Z, Bao J. Prognostic significance of pretreatment controlling nutritional status score in urological cancers: a systematic review and meta-analysis. Cancer Cell Int

2021;21(1):126.

21. Liu H, Yang XC, Liu DC, Tong C, Wen W, Chen RH. Clinical significance of the controlling nutritional status (CONUT) score in gastric cancer patients: A meta-analysis of 9,764 participants. Front Nutr. 2023;10:1156006.

22. Yin J, Qu J, Liang XX, Wang MM. Prognostic significance of controlling nutritional status score for patients with gastric cancer: A systematic review and meta-analysis. Exp Ther Med. 2023;25(5):202.

23. Takagi K, Domagala P, Polak WG, Buettner S, Wijnhoven BPL, Ijzermans JNM. Prognostic significance of the controlling nutritional status (CONUT) score in patients undergoing gastrectomy for gastric cancer: a systematic review and meta-analysis. BMC Surg. 2019;19(1):129.

24. Liu ZR, Zhou HG, Zhou Y, Yu ML, Cheng YL, Li J. Prognostic impact of the Controlling Nutritional Status Score in patients with biliary tract cancer: a systematic review and meta-analysis. Front Oncol. 2023;13:1240008.

25. Jiang H, Wang Z. Prognostic role of the controlling nutritional status (CONUT) score in patients with biliary tract cancer: a meta-analysis. Ann Med. 2023;55(2):2261461.

26. Chen J, Song P, Peng Z, Liu Z, Yang L, Wang L, et al. The Controlling Nutritional Status (CONUT) Score and Prognosis in Malignant Tumors: A Systematic Review and Meta-Analysis. Nutr Cancer. 2022;74(9):3146-63.

27. Chen J, Cao D, Peng Z, Song P, Liu Z, Yang L, et al. The prognostic value of the Controlling Nutritional Status score on patients undergoing nephrectomy for upper tract urothelial carcinoma or renal cell carcinoma: a systematic review and meta-analysis. Br J Nutr. 2022;128(2):217-24.

28. Xue W, Hu X, Zhang Y. The Association of Controlling Nutritional Status (CONUT) Score with Survival in Patients with Surgically Treated Renal Cell Carcinoma and Upper Tract Urothelial Carcinoma: A Systematic Review and Meta-Analysis. Nutr Cancer. 2022;74(6):1907-16.

29. Peng L, Meng C, Li J, You C, Du Y, Xiong W, et al. The prognostic significance of controlling nutritional status (CONUT) score for surgically treated renal cell cancer and upper urinary tract urothelial cancer: a systematic review and meta-analysis. Eur J Clin Nutr. 2022;76(6):801-10.

30. Shao J, Li J, Zhang XL, Wang G. Prognostic Significance of the Preoperative Controlled Nutritional Status Score in Lung Cancer Patients Undergoing Surgical Resection. Nutr Cancer. 2021;73(11-12):2211-8.

31. Zhang C, Li XK, Cong ZZ, Zheng C, Luo C, Xie K, et al. Controlling nutritional status is a prognostic factor for patients with lung cancer: a systematic review and meta-analysis. Ann Palliat Med. 2021;10(4):3896-905.

32. Takagi K, Buettner S, Ijzermans JNM. Prognostic significance of the controlling nutritional status (CONUT) score in patients with colorectal cancer: A systematic review and meta-analysis. Int J Surg. 2020;78:91-6.

33. Takagi K, Buettner S, Ijzermans JNM, Wijnhoven BPL. Systematic Review on the Controlling Nutritional Status (CONUT) Score in Patients Undergoing Esophagectomy for Esophageal Cancer. Anticancer Res. 2020;40(10):5343-9.

34. Lv J, Chen P, Wu J, Hu C. Prognostic value of pretreatment Controlling Nutritional Status score in esophageal cancer: a meta-analysis. Pathol Oncol Res. 2023;29:1611221.

35. Niu Z, Yan B. Prognostic and clinicopathological impacts of Controlling Nutritional Status (CONUT) score on patients with gynecological cancer: a meta-analysis. Nutr J. 2023;22(1):33.

36. Lu C, Chen Q, Fei L, Wang J, Wang C, Yu L. Prognostic impact of the controlling nutritional status score in patients with hematologic malignancies: A systematic review and meta-analysis. Front Immunol. 2022;13:952802.

37. Peng J, Hao Y, Rao B, Cao Y. Prognostic impact of the pre-treatment controlling nutritional status score in patients with non-small cell lung cancer: A meta-analysis. Medicine (Baltimore). 2021;100(26):e26488.

38. Li HY, Zhou P, Zhao YK, Ni HC, Luo XP, Li J. Prediction of all-cause mortality with malnutrition assessed by controlling nutritional status score in patients with heart failure: a systematic review and meta-analysis. Public Health Nutr. 2022;25(7):1799-806.

39. Peng J, Li XY, Huang MS, Ma MC, Huang Q, Huang N, et al. Prognostic value of prognostic nutritional index score and controlling nutritional status score in patients with glioblastoma: A comprehensive meta-analysis. Front Oncol. 2023;13:1117764.

40. Kazemian S, Tavolinejad H, Rashedi S, Yarahmadi P, Farrokhpour H, Kolte D. Meta-Analysis on the Association Between Nutritional Status and Outcomes After Transcatheter Aortic Valve Implantation. Am J Cardiol. 2023;186:109-16.

41. Ni J, Fang Y, Zhang J, Chen X. Predicting prognosis of heart failure using common malnutrition assessment tools: A systematic review and meta-analysis. Scott Med J. 2022;67(4):157-70.

42. Peng P, Chen L, Shen Q, Xu Z, Ding X. Prognostic Nutritional Index (PNI) and Controlling Nutritional Status (CONUT) score for predicting outcomes of breast cancer: A systematic review and meta-analysis. Pak J Med Sci. 2023;39(5):1535-41.

43. Takagi K, Domagala P, Polak WG, Buettner S, Ijzermans JNM. The Controlling Nutritional Status Score and Postoperative Complication Risk in Gastrointestinal and Hepatopancreatobiliary Surgical Oncology: A Systematic Review and Meta-Analysis. Ann Nutr Metab. 2019;74(4):303-12.

44. Zhang Y, Zhang X. Controlling nutritional status score, a promising prognostic marker in patients with gastrointestinal cancers after surgery: A systematic review and meta-analysis. Int J Surg. 2018;55:39-45.

45. Li L, Shou L. Prognostic and clinicopathological significance of the Controlling Nutritional Status (CONUT) score in patients with lymphoma: a meta-analysis. BMJ Open. 2024;14(3):e078320.

46. Jiao H, Wang L, Zhou X, Wu J, Li T. Prognostic Ability of Nutritional Indices for Outcomes of Bladder Cancer: A Systematic Review and Meta-Analysis. Urol Int. 2023;107(9):886-94.

47. Huang LJ, He RL, Sun XJ, Lv J, Chen SX. Association of Controlling Nutritional Status Score With Adverse Outcomes in Patients With Coronary Artery Disease: A Systematic Review and Meta-Analysis. Angiology. 2023;74(2):149-58.

48. Yan CK, Xie YY, Hua YQ, Li SH, Fu HX, Cheng ZQ, et al. Prognostic value of geriatric nutritional risk index in patients with diffuse large B-cell lymphoma: a meta-analysis. Clin Transl Oncol. 2023;26(2):515-23.

49. Cao D, Zhang ZX. Prognostic and clinicopathological role of geriatric nutritional risk index in patients with diffuse large B-cell lymphoma: A meta-analysis. Front Oncol. 2023;13:1169749.

50. Shen F, Ma Y, Guo W, Li F. Prognostic Value of Geriatric Nutritional Risk Index for Patients with Non-Small Cell Lung Cancer: A Systematic Review and Meta-Analysis. Lung. 2022;200(5):661-9.

51. Yang M, Liu Z, Li G, Li B, Li C, Xiao L, et al. Geriatric Nutritional Risk Index as a Prognostic Factor of Patients with Non-Small Cell Lung Cancer: A Meta-Analysis. Horm Metab Res. 2022;54(9):604-12.

52. Yiu CY, Liu CC, Wu JY, Tsai WW, Liu PH, Cheng WJ, et al. Efficacy of the Geriatric Nutritional Risk Index for Predicting Overall Survival in Patients with Head and Neck Cancer: A Meta-Analysis. Nutrients. 2023;15(20):4348.

53. Mao Y, Lan J. Prognostic value of the geriatric nutritional index in colorectal cancer patients undergoing surgical intervention: A systematic review and meta-analysis. Front Oncol. 2022;12:1066417.

54. Yuan F, Yuan Q, Hu J, An J. Prognostic Role of Pretreatment Geriatric Nutritional Risk Index in Colorectal Cancer Patients: A Meta-Analysis. Nutr Cancer. 2023;75(1):276-85.

55. Xu J, Sun Y, Gong D, Fan Y. Predictive Value of Geriatric Nutritional Risk Index in Patients with Colorectal Cancer: A Meta-Analysis. Nutr Cancer. 2023;75(1):24-32.

56. Zhao HM, Xu L, Tang P, Guo R. Geriatric Nutritional Risk Index and Survival of Patients With Colorectal Cancer: A Meta-Analysis. Front Oncol. 2022;12:906711.

57. Wu Q, Ye FG. Prognostic impact of geriatric nutritional risk index on patients with urological cancers: A meta-analysis. Front Oncol. 2023;12:1077792.

58. He L, Li Y, Qu LL, Zhang F. Prognostic and clinicopathological value of the geriatric nutritional risk index in gastric cancer: A meta-analysis of 5,834 patients. Front Surg. 2023;9:1087298.

59. Lu W, Shen J, Zou DH, Li P, Liu XC, Jian Y. Predictive role of preoperative geriatric nutritional risk index for clinical outcomes in surgical gastric cancer patients: A meta-analysis. Front Surg. 2022;9:1020482.

60. Zhang Q, Zhang L, Jin Q, He Y, Wu M, Peng H, et al. The Prognostic Value of the GNRI in Patients with Stomach Cancer Undergoing Surgery. J Pers Med. 2023;13(1):155.

61. Wang HY, Li C, Yang RY, Jin J, Liu D, Li WM. Prognostic Value of the Geriatric Nutritional Risk Index in Non-Small Cell Lung Cancer Patients: A Systematic Review and Meta-Analysis. Front Oncol. 2022;18:794862.

62. Wang Y, Luo L, Li J, Wang Y, Che G, Xie X. Prognostic Value of Pretreatment Geriatric Nutrition Risk Index in Lung Cancer Patients: A Meta-Analysis. Nutr Cancer. 2022;74(9):3164-71.

63. Xie HL, Tang SY, Wei LS, Gan JL. Geriatric nutritional risk index as a predictor of complications and long-term outcomes in patients with gastrointestinal malignancy: a systematic review and meta-analysis. Cancer Cell Int. 2020;20(1):530.

64. Fan Y, He L, Zhou YJ, Man CF. Predictive Value of Geriatric Nutritional Risk Index in Patients With Coronary Artery Disease: A Meta-Analysis. Front Nutr. 2021;8:736884.

65. Liu GD, Zou C, Jie Y, Wang P, Wang XY, Fan Y. Predictive Value of Geriatric Nutritional Risk Index in Patients With Lower Extremity Peripheral Artery Disease: A Meta-Analysis. Front Nutr. 2022;9:903293.

66. Zhou JF, Fang PH, Li XK, Luan SY, Xiao X, Gu YM, et al. Prognostic Value of Geriatric Nutritional Risk Index in Esophageal Carcinoma: A Systematic Review and Meta-Analysis. Front Nutr. 2022;9:831283.

67. Yang Q, Shen A, Chen X, Guo L, Peng H, Gao M. Clinical Significance of Nutrition and Inflammation in Esophageal Cancer Patients with Surgery: A Meta-Analysis. Nutr Cancer. 2022;74(9):3128-39.

68. Yu J, Zhang W, Wang C, Hu Y. The Prognostic Value of Pretreatment Geriatric Nutritional Risk Index in Esophageal Cancer: A Meta-Analysis. Nutr Cancer. 2022;74(9):3202-10.

69. Fan H, Ma W, Fu Y, Yi T, Tian J. Association of Geriatric Nutritional Risk Index with Survival Outcomes in Patients with Esophageal Squamous Cell Carcinoma: A Meta-Analysis. Nutr Cancer. 2022;74(8):2796-802.

70. Li H, Cen K, Sun W, Feng B. Prognostic value of geriatric nutritional risk index in elderly patients with heart failure: a meta-analysis. Aging Clin Exp Res. 2021;33(6):1477-86.

71. Lv GY, An L, Sun DW. Geriatric Nutritional Risk Index Predicts Adverse Outcomes in Human Malignancy: A Meta-Analysis. Dis Markers. 2019;2019:4796598.

72. YUAN Na LF, LIU Huan-bing. GNRI can predict the outcomes of maintenance hemodialysis patients—a meta-analysis. Chin J Blood Purif. 2021;20(8):516-20+35.

73. Li L, He J. Prognostic Role of Geriatric Nutritional Risk Index in Patients with Pancreatic Cancer: A Meta-Analysis. Nutr Cancer. 2023;75(7):1531-40.

74. Yu Q, Tian M, Pi G, Jia Y, Jin X. Geriatric nutritional risk index as a predictor of prognosis in hematologic malignancies: a systematic review and meta-analysis. Front Nutr. 2023;10:1274592.

75. Liu W, Li M, Lian S, Hou X, Ling Y. Geriatric nutritional risk index as a predictor for postoperative complications in patients with solid cancers: a meta-analysis. Front Oncol. 2024;14:1266291.

76. Luan C, Wang F, Wei N, Chen B. Prognostic nutritional index and the prognosis of diffuse large b-cell lymphoma: A meta-analysis. Cancer Cell Int. 2020;20(1):455.

77. Jiang AM, Zhao R, Liu N, Ma YY, Ren MD, Tian T, et al. The prognostic value of pretreatment prognostic nutritional index in patients with small cell lung cancer and it's influencing factors: A meta-analysis of observational studies. J Thorac Dis. 2020;12(10):5718-28.

78. Ren W, Wang H, Xiang T, Liu G. Prognostic Role of Preoperative Onodera’s Prognostic Nutritional Index (OPNI) in Gastrointestinal Stromal Tumors: a Systematic Review and Meta-analysis. J Gastrointest Cancer. 2023;54(3):731-8.

79. Kang N, Gu H, Ni Y, Wei X, Zheng S. Prognostic and clinicopathological significance of the Prognostic Nutritional Index in patients with gastrointestinal stromal tumours undergoing surgery: a meta-analysis. BMJ Open. 2022;12(12):e064577.

80. Li Z, Zhang D, Mo C, Zhu P, Fan X, Tang T. The prognostic significance of prognostic nutritional index in gastrointestinal stromal tumors: A systematic review and meta-analysis. Medicine (Baltimore). 2022;101(47):e32067.

81. Hu Y, Shen J, Liu R, Feng Z, Zhang C, Ling L, et al. Prognostic value of pretreatment prognostic nutritional index in non-small cell lung cancer: A systematic review and meta-analysis. Int J Biol Markers. 2018;33(4):372-8.

82. Wang Z, Wang Y, Zhang X, Zhang T. Pretreatment prognostic nutritional index as a prognostic factor in lung cancer: Review and meta-analysis. Clin Chim Acta. 2018;486:303-10.

83. Zhang Q, Bao J, Zhu ZY, Jin MX. Prognostic nutritional index as a prognostic factor in lung cancer patients receiving chemotherapy: a systematic review and meta-analysis. Eur Rev Med Pharmacol Sci. 2021;25(18):5636-52.

84. Wang DP, Kang K, Lin Q, Hai J. Prognostic Significance of Preoperative Systemic Cellular Inflammatory Markers in Gliomas: A Systematic Review and Meta-Analysis. Clin Transl Sci. 2020;13(1):179-88.

85. Liu M, Wang L. Prognostic significance of preoperative serum albumin, albumin-to-globulin ratio, and prognostic nutritional index for patients with glioma: A meta-analysis. Medicine (Baltimore). 2020;99(27):e20927.

86. Hung KC, Sun CK, Chang YP, Wu JY, Huang PY, Liu TH, et al. Association of prognostic nutritional index with prognostic outcomes in patients with glioma: a meta-analysis and systematic review. Front Oncol. 2023;13:1188292.

87. Li J, Xu R, Hu DM, Zhang Y, Gong TP, Wu XL. Prognostic Nutritional Index Predicts Outcomes of Patients after Gastrectomy for Cancer: A Systematic Review and Meta-Analysis of Nonrandomized Studies. Nutr Cancer. 2019;71(4):557-68.

88. Yang Y, Gao P, Song Y, Sun J, Chen X, Zhao J, et al. The prognostic nutritional index is a predictive indicator of prognosis and postoperative complications in gastric cancer: A meta-analysis. Eur J Surg Oncol. 2016;42(8):1176-82.

89. Lv X, Zhang Z, Yuan W. Pretreatment Prognostic Nutritional Index (PNI) as a Prognostic Factor in Patients with Biliary Tract Cancer: A Meta-Analysis. Nutr Cancer. 2021;73(10):1872-81.

90. Qi F, Zhou X, Wang Y, Wang Y, Wang Y, Zhang Q, et al. Pre-treatment prognostic nutritional index may serve as a potential biomarker in urinary cancers: a systematic review and meta-analysis. Cancer Cell Int. 2018;18:207.

91. Sun G, Li Y, Peng Y, Lu D, Zhang F, Cui X, et al. Impact of the preoperative prognostic nutritional index on postoperative and survival outcomes in colorectal cancer patients who underwent primary tumor resection: a systematic review and meta-analysis. Int J Colorectal Dis. 2019;34(4):681-9.

92. Yang Y, Gao P, Chen X, Song Y, Shi J, Zhao J, et al. Prognostic significance of preoperative prognostic nutritional index in colorectal cancer: results from a retrospective cohort study and a meta-analysis. Oncotarget. 2016;7(36):58543-52.

93. Li S, Tian G, Chen Z, Zhuang Y, Li G. Prognostic Role of the Prognostic Nutritional Index in Pancreatic Cancer: A Meta-analysis. Nutr Cancer. 2019;71(2):207-13.

94. Zhang X, Su Y. Low Prognostic Nutritional Index Predicts Adverse Outcomes in Patients With Heart Failure: A Systematic Review and Meta-analysis. Angiology. 2023;75(4):305-13.

95. Chen MY, Wen JX, Lu MT, Jian XY, Wan XL, Xu ZW, et al. Association Between Prognostic Nutritional Index and Prognosis in Patients With Heart Failure: A Meta-Analysis. Front Cardiovasc Med. 2022;9:918566.

96. Chang WT, Sun CK, Wu JY, Yu CH, Chang YJ, Lin MC, et al. Association of prognostic nutritional index with long-term mortality in patients receiving percutaneous coronary intervention for acute coronary syndrome: a meta-analysis. Sci Rep. 2023;13(1):13102.

97. Shi Y, Zhang Y, Niu Y, Chen Y, Kou C. Prognostic role of the prognostic nutritional index (PNI) in patients with head and neck neoplasms undergoing radiotherapy: A meta-analysis. PLoS One. 2021;16(9):e0257425.

98. Luan CW, Tsai YT, Yang HY, Chen KY, Chen PH, Chou HH. Pretreatment prognostic nutritional index as a prognostic marker in head and neck cancer: a systematic review and meta-analysis. Sci Rep. 2021;11(1):17117.

99. Hu G, Ding Q, Zhong K, Wang S, Wang S, Huang L. Low pretreatment prognostic nutritional index predicts poor survival in breast cancer patients: A meta-analysis. PLoS One. 2023;18(1):e0280669.

100. Meng C, Gan L, Li K, Yi F, Peng L, Li J, et al. Prognostic nutritional index before surgical treatment may serve as a prognostic biomarker for patients with upper tract urothelial carcinoma: A systematic review and meta-analysis. Front Nutr. 2022;9:972034.

101. Dai Y, Liu M, Lei L, Lu S. Prognostic significance of preoperative prognostic nutritional index in ovarian cancer: A systematic review and meta-analysis. Medicine (Baltimore). 2020;99(38):e21840.

102. Tan X, Chen H. The Prognostic Value of Prognostic Nutritional Index in Patients with Ovarian Cancer: A Systematic Review and Meta-Analysis. Nutr Cancer. 2023;75(1):73-81.

103. Fan X, Chen G, Li Y, Shi Z, He L, Zhou D, et al. The Preoperative Prognostic Nutritional Index in Hepatocellular Carcinoma After Curative Hepatectomy: A Retrospective Cohort Study and Meta-Analysis. J Invest Surg. 2021;34(8):826-33.

104. Man Z, Pang Q, Zhou L, Wang Y, Hu X, Yang S, et al. Prognostic significance of preoperative prognostic nutritional index in hepatocellular carcinoma: a meta-analysis. HPB (Oxford). 2018;20(10):888-95.

105. Liao G, Zhao Z, Yang H, Chen M, Li X. Can Prognostic Nutritional Index be a Prediction Factor in Esophageal Cancer?: A Meta-Analysis. Nutr Cancer. 2020;72(2):187-93.

106. Hao J, Chen C, Wan F, Zhu Y, Jin H, Zhou J, et al. Prognostic Value of Pre-Treatment Prognostic Nutritional Index in Esophageal Cancer: A Systematic Review and Meta-Analysis. Front Oncol. 2020;10:797.

107. Xue Y, Zhou X, Xue L, Zhou R, Luo J. The role of pretreatment prognostic nutritional index in esophageal cancer: A meta-analysis. J Cell Physiol. 2019;234(11):19655-62.

108. Li P, Wang X, Lai Y, Zhou K, Tang Y, Che G. The prognostic value of pre-treatment prognostic nutritional index in esophageal squamous cell carcinoma: A meta-analysis. Medicine (Baltimore). 2019;98(22):e15280.

109. Mao C, Xu W, Ma W, Wang C, Guo Z, Yan J. Prognostic Value of Pretreatment Prognostic Nutritional Index in Patients With Renal Cell Carcinoma: A Meta-Analysis. Front Oncol. 2021;11:719941.

110. Kim SI, Kim SJ, Kim SJ, Cho DS. Prognostic nutritional index and prognosis in renal cell carcinoma: A systematic review and meta-analysis. Urol Oncol. 2021;39(10):623-30.

111. Xiong SC, Hu X, Lia T, Wang YH, Li X. Prognostic Significance of Prognostic Nutritional Index in Patients with Renal Cell Carcinoma: A Meta-Analysis. Nutr Cancer. 2022;74(3):860-8.

112. Peng Q, Liu L, Li T, Lei C, Wan H. Prognostic impact of prognostic nutritional index on renal cell carcinoma: A meta-analysis of 7,629 patients. PLoS One. 2022;17(3):e0265119.

113. Xue S, Zhao H, Zhang K, Zhang H, Wang W. Prognostic and Clinicopathological Correlations of Pretreatment Prognostic Nutritional Index in Renal Cell Carcinoma: A Meta-Analysis. Urol Int. 2022;106(6):567-80.

114. Wang X, Wang Y. The prognostic nutritional index is prognostic factor of gynecological cancer: A systematic review and meta-analysis. Int J Surg. 2019;67:79-86.

115. Zheng Y, Wang K, Ou Y, Hu X, Wang Z, Wang D, et al. Prognostic value of a baseline prognostic nutritional index for patients with prostate cancer: a systematic review and meta-analysis. Prostate Cancer Prostatic Dis. 2023;27(4):604-13.

116. Dai M, Sun Q. Prognostic and clinicopathological significance of prognostic nutritional index (PNI) in patients with oral cancer: a meta-analysis. Aging (Albany NY). 2023;15(5):1615-27.

117. Tang M, Jia Z, Zhang J. The prognostic role of prognostic nutritional index in nasopharyngeal carcinoma: A systematic review and meta-analysis. Int J Clin Oncol. 2021;26(1):66-77.

118. Tu X, Ren J, Zhao Y. Prognostic value of prognostic nutritional index in nasopharyngeal carcinoma: A meta-analysis containing 4511 patients. Oral Oncol. 2020;110:104991.

119. Gao QL, Shi JG, Huang YD. Prognostic Significance of Pretreatment Prognostic Nutritional Index (PNI) in Patients with Nasopharyngeal Carcinoma: A Meta-Analysis. Nutr Cancer. 2021;73(9):1657-67.

120. Zhang S, Wang H, Chen S, Cai S, Zhou S, Wang C, et al. Prognostic nutritional index and prognosis of patients with coronary artery disease: A systematic review and meta-analysis. Front Nutr. 2023;10:1114053.

121. Sun K, Chen S, Xu J, Li G, He Y. The prognostic significance of the prognostic nutritional index in cancer: a systematic review and meta-analysis. J Cancer Res Clin Oncol. 2014;140(9):1537-49.

122. Bullock AF, Greenley SL, McKenzie GAG, Paton LW, Johnson MJ. Relationship between markers of malnutrition and clinical outcomes in older adults with cancer: systematic review, narrative synthesis and meta-analysis. Eur J Clin Nutr. 2020;74(11):1519-35.

123. Guo H, Yang L, Liu J, Yu X, Chen L, Huang Y. Prognostic Nutritional Index and the Risk of Postoperative Complications After Spine Surgery: A Meta-Analysis. World Neurosurg. 2024;185:e572-e81.

124. Zhao P, Wu Z, Wang Z, Wu C, Huang X, Tian B. Prognostic role of the prognostic nutritional index in patients with pancreatic cancer who underwent curative resection without preoperative neoadjuvant treatment: A systematic review and meta-analysis. Front Surg. 2022;9:992641.

125. Liu CC, Liu PH, Chen HT, Chen JY, Lee CW, Cheng WJ, et al. Association of Preoperative Prognostic Nutritional Index with Risk of Postoperative Acute Kidney Injury: A Meta-Analysis of Observational Studies. Nutrients. 2023;15(13):2929.

126. Li P, Lai Y, Tian L, Zhou Q. The prognostic value of prognostic nutritional index in advanced cancer receiving PD-1/L1 inhibitors: A meta-analysis. Cancer Med. 2022;11(16):3048-56.

127. Ni L, Huang J, Ding J, Kou J, Shao T, Li J, et al. Prognostic Nutritional Index Predicts Response and Prognosis in Cancer Patients Treated With Immune Checkpoint Inhibitors: A Systematic Review and Meta-Analysis. Front Nutr. 2022;9:823087.

128. Yan X, Wang J, Mao J, Wang Y, Wang X, Yang M, et al. Identification of prognostic nutritional index as a reliable prognostic indicator for advanced lung cancer patients receiving immune checkpoint inhibitors. Front Nutr. 2023;10:1213255.

129. Xu XT, Qian Y, Tian MX, Ding CC, Guo H, Tang J, et al. Predictive Impact of Prognostic Nutritional Index in Patients with Cancer Treated with Immune Checkpoint Inhibitors: A Systematic Review and Meta-Analysis. Nutr Cancer. 2023;75(6):1413-26.

130. Zhang L, Ma W, Qiu Z, Kuang T, Wang K, Hu B, et al. Prognostic nutritional index as a prognostic biomarker for gastrointestinal cancer patients treated with immune checkpoint inhibitors. Front Immunol. 2023;14:1219929.

131. Zheng WH, Zhao YH, Yao Y, Huang HB. Prognostic role of bioelectrical impedance phase angle for critically ill patients: A systemic review and meta-analysis. Front Med (Lausanne). 2023;9:1059747.

132. Arab A, Karimi E, Vingrys K, Shirani F. Is phase angle a valuable prognostic tool in cancer patients' survival? A systematic review and meta-analysis of available literature. Clin Nutr. 2021;40(5):3182-90.

133. Fernández-Jiménez R, Martín-Masot R, Cornejo-Pareja I, Vegas-Aguilar IM, Herrador-López M, Tinahones FJ, et al. Phase angle as a marker of outcome in hospitalized pediatric patients. A systematic review of the evidence (GRADE) with meta-analysis. Rev Endocr Metab Disord. 2023;24(4):751-65.

134. Ahmadi SF, Zahmatkesh G, Streja E, Mehrotra R, Rhee CM, Kovesdy CP, et al. Association of body mass index with mortality in peritoneal dialysis patients: A systematic review and meta-analysis. Perit Dial Int. 2016;36(3):315-25.

135. De Paola L, Mehta A, Pana TA, Carter B, Soiza RL, Kafri MW, et al. Body Mass Index and Mortality, Recurrence and Readmission after Myocardial Infarction: Systematic Review and Meta-Analysis. J Clin Med. 2022;11(9).

136. Cao C, Wang R, Wang JM, Bunjhoo H, Xu YJ, Xiong WN. Body Mass Index and Mortality in Chronic Obstructive Pulmonary Disease: A Meta-Analysis. PLOS ONE. 2012;7(8):e43892.

137. Simillis C, Taylor B, Ahmad A, Lal N, Afxentiou T, Powar MP, et al. A systematic review and meta-analysis assessing the impact of body mass index on long-term survival outcomes after surgery for colorectal cancer. Eur J Cancer. 2022;172:237-51.

138. He X, Ji J, Liu C, Luo Z, Tang J, Yan H, et al. Body mass index and weight loss as risk factors for poor outcomes in patients with idiopathic pulmonary fibrosis: a systematic review and meta-analysis. Ann Med. 2024;56(1):2311845.
