# Supplementary material for: Association between malnutrition diagnosed by different screening and assessment tools and clinical outcomes: an umbrella review
Source: Front Nutr. 2025 Oct 9;12:1676201. doi: 10.3389/fnut.2025.1676201 (PMC12545068; doi:10.3389/fnut.2025.1676201)
Supplement: Supplementary file 4 [file Table_4.DOCX]

**Supplementary Figure4:** Evaluation of bias and heterogeneity.

| First author (Ref) | NRTs/NSTs | outcomes | | Egger's p value | I^2^ (95% CI) | ESS |
| --- | --- | --- | --- | --- | --- | --- |
| Liu^1^ | **MNA-SF** |  | mortality after hip fracture | 0.039 | 84.8% | 0(0) |
| Hu^2^ | **low MNA-SF** in HF |  | all-cause mortality | 0.180 | 0.0% | 0(0) |
| Lin^3^ | **MNA-SF** in HF |  | mortality | U | 0.0% | 0(0) |
| Osório^4^ | **MNA-SF in patients hospitalized with decompensated HF** |  | all-cause mortality | U | 0.0% | 0(0) |
| Hu^2^ | **SGA** in HF |  | all-cause mortality | 0.915 | 0.0% | 0(0) |
| Peng^5^ | **GLIM** in patients with cancer | multivariate regression model | malnourished, OS | 0.010 | 70.2% | 1(1) |
|  |  |  | moderately malnourished, OS | 0.028 | 31.4% | 0(0) |
|  |  |  | severely malnourished, OS | 0.235 | 52.7% | 0(0) |
|  |  |  | DFS | 0.025 | 46.8% | 0(1) |
|  |  |  | overall complications | U | 25.3% | 0(0) |
|  |  | univariate regression model | malnourished, OS | 0.050 | 69.3% | 0(1) |
|  |  |  | moderately malnourished, OS | 0.222 | 53.7% | 0(0) |
|  |  |  | severely malnourished, OS | 0.273 | 76.3% | 0(0) |
|  |  | POCs | overall complications | 0.647 | 80.6% | 0(0) |
|  |  |  | Clavien-Dindo grade≥IIa | 0.180 | 0.0% | 0(0) |
|  |  |  | Clavien-Dindo grade≥IIIa | 0.842 | 85.1% | 1(1) |
| Ryota^6^ | **GLIM** in patients with cancer |  | OS | 0.021 | 36.7% | 1(1) |
|  |  |  | RFS | U | 62.9% | 0(0) |
|  |  | POCs | total number of POCs | 0.935 | 87% | 0(1) |
|  |  |  | severe complications | 0.921 | 92.3% | 0(1) |
|  |  |  | infectious complications | 0.491 | 61.9% | 0(0) |
|  |  |  | anastomotic leakage | 0.028 | 0.0% | 0(0) |
|  |  |  | postoperative pneumonia | 0.454 | 0.0% | 0(0) |
| Xu^7^ | **GLIM** in patients with cancer |  | OS | 0.005 | 57.9% | 1(1) |
|  |  |  | DFS | 0.025 | 46.8% | 0(1) |
| Yin^8^ | **GLIM** in patients with cancer | malnutrition, univariate | OS | 0.843 | 0.0% | 0(0) |
|  |  | moderate malnutrition, univariate | OS | 0.964 | 31.4% | 0(0) |
|  |  | severe malnutrition, univariate | OS | 0.486 | 0.0% | 0(0) |
|  |  | malnutrition, multivariate | OS | 0.975 | 0.0% | 0(0) |
|  |  | moderate malnutrition, multivariate | OS | 0.383 | 0.0% | 0(0) |
|  |  | severe malnutrition, multivariate | OS | 0.175 | 0.0% | 0(0) |
| Lidoriki^9^ | GLIM in esophageal and gastric cancer patients |  | overall POCs | 0.266 | 88.0% | 0(1) |
|  |  |  | OS | 0.057 | 31.8% | 0(0) |
|  |  |  | DFS | 0.026 | 2.1% | 0(0) |
| Sun^10^ | **NRS 2002 in patients undergoing abdominal surgery** |  | overall complications | 0.149 | 1.5% | 0(0) |
|  |  |  | infective complications | 0.193 | 0.0% | 0(0) |
|  |  |  | mortality | 0.066 | 0.0% | 0(0) |
|  |  |  | length of hospital stay | 0.290 | 89.7% | 0(0) |
| Zang^11^ | **NRS 2002** in patients with cancer |  | OS | 0.025 | 68.5% | 0(0) |
|  |  |  | POCs | 0.181 | 0.0% | 0(1) |
| Chen^12^ | NPS in patients with GI cancers |  | OS | 0.380 | 61.9% | 0(0) |
|  |  |  | CSS | 0.235 | 41.6% | 0(1) |
|  |  |  | RFS/PFS | 0.411 | 39.6% | 0(0) |
| WANG^13^ | NPS in LC |  | OS | 0.036 | 65.2% | 0(1) |
|  |  |  | DFS | 0.003 | 62.3% | 0(0) |
| Wu^14^ | mGPS in PC |  | OS | 0.196 | 65.0% | 1(1) |
| Nie^15^ | mGPS **in gynecologic cancer** |  | OS | 0.361 | 20.9% | 0(0) |
|  |  |  | PFS | U | 29.2% | 0(0) |
|  | GPS **in gynecologic cancer** |  | OS | 0.029 | 40.2% | 0(0) |
|  |  |  | PFS | 0.192 | 29.9% | 0(1) |
| Hu^16^ | mGPS in RCC |  | OS | 0.140 | 60.6% | 0(0) |
|  |  |  | CSS | 0.787 | 0.0% | 0(0) |
|  |  |  | RFS | 0.464 | 0.0% | 0(0) |
|  |  |  | PFS | U | 0.0% | 0(0) |
| Zhou^17^ | mGPS in BTC |  | OS | 0.109 | 86.8% | 1(1) |
|  |  |  | DFS/RFS | 0.988 | 39.2% | 0(1) |
| Zhang^18^ | MNA **in older adults with cancer** |  | OS | 0.864 | 73.7% | 1(1) |
| Hu^2^ | low MNA in HF |  | all-cause mortality | 0.225 | 72.5% | 0(1) |
| Lin^3^ | MNA in HF |  | mortality | 0.214 | 0.0% | 0(0) |
| Ma^19^ | CONUT in PC |  | OS | 0.234 | 88.9% | 0(0) |
|  |  |  | RFS | 0.868 | 85.5% | 0(0) |
| Niu^20^ | high CONUT score in UCs |  | OS | 0.033 | 45.2% | 1(1) |
|  |  |  | CSS | 0.036 | 51.4% | 0(1) |
|  |  |  | RFS/DFS/PFS | 0.003 | 19.4% | 0(1) |
| Liu^21^ | CONUT in GC |  | OS | 0.570 | 32.9% | 1(1) |
|  |  |  | RFS | 0.004 | 29.7% | 0(0) |
|  |  |  | POCs | 0.179 | 69.0% | 0(1) |
| Yin^22^ | CONUT in GC | high CONUT score group compared with low CONUT score group | OS | 0.194 | 11.9% | 0(1) |
|  |  |  | RFS | 0.038 | 17.6% | 1(1) |
|  |  |  | CSS | 0.360 | 73.2% | 0(0) |
|  |  |  | risk of complication | 0.002 | 67.6% | 1(1) |
| Takagi^23^ | high CONUT group versus low CONUT group in GC |  | OS | 0.305 | 53.6% | 0(0) |
|  |  |  | CSS | 0.098 | 63.5% | 0(0) |
|  |  |  | RFS | 0.047 | 16.0% | 0(1) |
| Liu^24^ | CONUT in BTC |  | OS | 0.012 | 53.6% | 0(1) |
|  |  |  | RFS | 0.321 | 43.5% | 0(1) |
| Jiang^25^ | CONUT in BTC |  | OS | 0.000 | 80.0% | 1(1) |
|  |  |  | RFS | 0.045 | 49.2% | 0(1) |
| Chen^26^ | high CONUT score **in various malignant tumors** |  | OS | 0.000 | 67.1% | 1(1) |
|  |  |  | CSS | 0.000 | 49.1% | 0(1) |
|  |  |  | PFS/RFS | 0.021 | 73.3% | 1(1) |
|  |  |  | DFS | 0.001 | 28.5% | 0(1) |
|  |  |  | POCs | 0.649 | 63.4% | 1(1) |
|  |  |  | mortality | 0.118 | 0.0% | 0(0) |
| Chen^27^ | low CONUT vs. high CONUT score in UTUC/RCC | UTUC | OS | 0.414 | 73.8% | 0(1) |
|  |  |  | CSS | 0.308 | 78.9% | 0(0) |
|  |  |  | DFS | 0.223 | 3.0% | 0(0) |
|  |  | RCC | OS | 0.960 | 0.0% | 0(0) |
|  |  |  | CSS | 0.428 | 0.0% | 0(0) |
|  |  |  | DFS | U | 11.3% | 0(1) |
| Xue^28^ | high CONUT score vs. low CONUT score in surgically-treated RCC or UTUC |  | RFS | 0.087 | 62.2% | 0(0) |
|  |  |  | CSS | 0.009 | 86.6% | 0(0) |
|  |  |  | OS | 0.035 | 82.3% | 0(0) |
| Peng^29^ | CONUT in UTUC and RCC |  | OS | 0.006 | 65.8% | 1(1) |
|  |  |  | CSS | 0.001 | 60.2% | 0(1) |
|  |  |  | RFS | 0.210 | 53.5% | 1(1) |
|  |  |  | DFS | U | 0.0% | 0(0) |
| Shao^30^ | CONUT in LC |  | OS | 0.183 | 7.4% | 0(0) |
|  |  |  | DFS | 0.055 | 10.2% | 0(0) |
| Zhang^31^ | high CONUT group vs. low CONUT group in LC |  | OS | 0.023 | 0.0% | 0(0) |
|  |  |  | DFS | 0.014 | 0.0% | 0(1) |
|  |  |  | CSS | U | 0.0% | 0(0) |
|  |  |  | PFS | U | 80.6% | 0(0) |
| Takagi^32^ | low CONUT vs. high CONUT score in CRC |  | OS | 0.714 | 29.7% | 0(0) |
|  |  |  | CSS | U | 0.0% | 0(0) |
|  |  |  | RFS | 0.761 | 0.0% | 0(0) |
| TAKAGI^33^ | CONUT in EC |  | OS | 0.498 | 0.0% | 0(0) |
|  |  |  | CSS | U | 25.8% | 0(0) |
|  |  |  | RFS | U | 0.0% | 0(0) |
| Lv^34^ | CONUT in EC |  | OS | 0.041 | 59.4% | 1(1) |
|  |  |  | PFS | 0.602 | 19.9% | 0(0) |
|  |  |  | CSS | 0.860 | 0.0% | 0(0) |
| Niu^35^ | COUNT in gynecological cancer |  | OS | 0.226 | 57.4% | 0(1) |
|  |  |  | PFS | 0.635 | 0.0% | 0(0) |
| Lu^36^ | high CONUT score in hematologic malignancy |  | OS | 0.038 | 58.1% | 0(0) |
|  |  |  | PFS | U | 0.0% | 0(0) |
| Peng^37^ | high CONUT score in NSCLC |  | OS | 0.001 | 31.6% | 0(1) |
|  |  |  | DFS/RFS | 0.257 | 6.7% | 1(1) |
|  |  |  | CSS | U | 47.3% | 0(0) |
| Li^38^ | CONUT in HF |  | all-cause mortality | 0.013 | 0.0% | 0(1) |
| Hu^2^ | high CONUT score in HF |  | all-cause mortality | 0.025 | 88.1% | 1(1) |
| Ni^39^ | CONUT in HF |  | mortality | 0.028 | 51.2% | 0(0) |
| Peng^40^ | CONUT in glioblastoma | univariate analysis | OS | 0.309 | 26.1% | 0(0) |
|  |  | multivariate analysis | OS | 0.036 | 38.7% | 0(1) |
| Osório^4^ | CONUT in patients hospitalized with decompensated HF |  | all-cause mortality | 0.363 | 91.2% | 0(1) |
| Kazemian^41^ | CONUT in patients who underwent TAVI |  | 1-year all-cause mortality | 0.097 | 83.6% | 0(0) |
| Peng^42^ | CONUT in BC |  | OS | U | 0.0% | 0(0) |
| Takagi^43^ | CONUT in GI and HPB cancers |  | mortality | 0.475 | 0.0% | 0(0) |
|  |  |  | major complications (CDc ≥3) | 0.777 | 78.5% | 1(1) |
|  |  |  | overall complications (CDc ≥2) | 0.204 | 2.2% | 0(0) |
| Zhang^44^ | CONUT in GI |  | OS | 0.412 | 0.0% | 0(1) |
|  |  |  | CSS | 0.580 | 0.0% | 0(0) |
|  |  |  | RFS | 0.068 | 0.0% | 0(1) |
| Li^45^ | CONUT in lymphoma |  | OS | 0.002 | 90.7% | 0(1) |
|  |  |  | PFS | 0.324 | 80.2% | 0(0) |
| Jiao^46^ | CONUT in bladder cancer |  | OS | 0.501 | 0.0% | 0(0) |
|  |  |  | RFS | 0.017 | 0.0% | 0(0) |
| Huang^47^ | CONUT in CAD | high vs low CONUT score | MACEs | 0.089 | 74.3% | 0(0) |
|  |  |  | all-cause mortality | 0.699 | 57.5% | 0(0) |
| Yan^48^ | GNRI in DLBCL |  | OS | 0.202 | 77.3% | 0(1) |
|  |  |  | PFS | 0.955 | 14.7% | 0(0) |
| Cao^49^ | GNRI in NSCLC |  | OS | 0.217 | 48.9% | 0(0) |
|  |  |  | PFS | U | 44.3% | 0(0) |
| Shen^50^ | GNRI in NSCLC |  | OS | 0.779 | 0.0% | 0(0) |
|  |  |  | DFS | 0.514 | 33.7% | 0(0) |
| Yang^51^ | GNRI in NSCLC |  | OS | 0.222 | 29.7% | 0(1) |
|  |  |  | PFS | 0.209 | 29.2% | 0(0) |
|  |  |  | CSS | U | 0.0% | 0(0) |
| Yiu^52^ | GNRI in HNC |  | OS | 0.032 | 58.4% | 0(0) |
|  |  |  | caner related diverse survival outcomes | 0.085 | 0.0% | 0(0) |
| Mao^53^ | GNRI in CRC |  | OS | 0.018 | 68.7% | 0(1) |
|  |  |  | DFS | 0.033 | 48.6% | 1(1) |
|  |  |  | complications | 0.567 | 0.0% | 0(0) |
| Yuan^54^ | GNRI in CRC |  | OS | 0.011 | 63.7% | 0(0) |
|  |  |  | DFS | 0.008 | 46.8% | 1(1) |
| Xu^55^ | GNRI in CRC | the low vs. high GNRI | OS | 0.021 | 67.2% | 0(0) |
|  |  |  | DFS | 0.008 | 46.3% | 1(1) |
|  |  | 98 | OS | 0.018 | 48.1% | 0(0) |
| Zhao^56^ | GNRI in CRC |  | OS | 0.008 | 60.8% | 0(0) |
|  |  |  | PFS | 0.009 | 33.1% | 1(1) |
| Wu^57^ | GNRI in UCs |  | OS | 0.882 | 75.6% | 0(1) |
|  |  |  | RFS/PFS | 0.068 | 0.0% | 0(0) |
|  |  |  | CSS | 0.548 | 80.0% | 0(1) |
| He^58^ | GNRI in GC |  | OS | 0.000 | 88.4% | 1(1) |
|  |  |  | CSS | 0.406 | 0.0% | 0(0) |
| Lu^59^ | GNRI in GC |  | OS | 0.016 | 88.8% | 0(1) |
|  |  |  | CSS | U | 0.0% | 0(0) |
| Zhang^60^ | GNRI in GC | dichotomous variable | OS | 0.825 | 0.0% | 0(0) |
|  |  | continuous variable | OS | 0.088 | 45.9% | 1(0) |
|  |  |  | CSS | 0.436 | 0.0% | 0(0) |
|  |  |  | POCs | 0.004 | 0.0% | 1(1) |
| Wang^61^ | GNRI in LC |  | OS | 0.042 | 24.7% | 0(0) |
|  |  |  | CSS | U | 0.0% | 0(0) |
|  |  |  | RFS | U | 54.6% | 0(0) |
| Wang^62^ | GNRI in LC |  | OS | 0.140 | 0.0% | 0(0) |
|  |  |  | RFS | 0.095 | 42.4% | 0(0) |
| Xie^63^ | GNRI in gastrointestinal malignancy |  | OS | 0.012 | 0.0% | 0(0) |
|  |  |  | complications | 0.260 | 0.0% | 0(1) |
|  |  |  | RFS | U | 0.0% | 0(0) |
|  |  |  | DFS | U | 0.0% | 1(0) |
|  |  |  | CSS | U | 0.0% | 1(0) |
| Fan^64^ | the lowest vs. the highest GNRI in CAD |  | all-cause mortality | 0.022 | 0.0% | 0(1) |
|  |  |  | MACE | 0.654 | 75.3% | 0(1) |
| Liu^65^ | GNRI in PAD |  | MACLEs | U | 0.0% | 0(0) |
|  |  |  | all-cause mortality | 0.686 | 0.0% | 0(1) |
| Zhou^66^ | GNRI in EC |  | OS | 0.742 | 0.0% | 0(1) |
|  |  |  | CSS | U | 0.0% | 0(0) |
| Yang^67^ | GNRI in EC |  | OS | 0.299 | 0.0% | 0(0) |
|  |  |  | CSS | U | 0.0% | 0(0) |
| Yu^68^ | GNRI in EC |  | OS | 0.007 | 49.0% | 1(1) |
|  |  |  | PFS | 0.007 | 66.6% | 0(1) |
| Fan^69^ | the lowest vs. the highest GNRI in ESCC |  | OS | 0.013 | 9.6% | 0(1) |
|  |  |  | CSS | U | 0.0% | 0(0) |
| LIU^1^ | GNRI |  | mortality after hip fracture | 0.067 | 86.7% | 0(0) |
| Hu^2^ | Low GNRI in HF |  | all-cause mortality | 0.000 | 90.8% | 1(1) |
| Lin^3^ | GNRI in HF |  | mortality | 0.009 | 70.7% | 0(1) |
| Ni^39^ | GNRI in HF |  | mortality | 0.567 | 95.6% | 0(0) |
| Li^70^ | GNRI in elderly patients with HF | the lowest versus the highest | all-cause mortality | 0.081 | 0.0% | 0(0) |
|  |  |  | major cardiovascular events | 0.047 | 83.7% | 0(1) |
| Osório^4^ | GNRI in patients hospitalized with decompensated HF |  | all-cause mortality | 0.029 | 80.9% | 1(1) |
| Lv^71^ | GNRI in malignancies |  | OS | 0.000 | 84.2% | 1(1) |
|  |  |  | CSS | 0.162 | 14.4% | 0(0) |
|  |  |  | DFS | 0.037 | 70.5% | 0(0) |
|  |  |  | PFS | U | 50.8% | 0(0) |
| Yuan^72^ | GNRI in maintenance hemodialysis patients | continuous variable | all-cause mortality | 0.874 | 83.1% | 1(0) |
|  |  | binary categorical variable | cardiovascular mortality | 0.108 | 15.1% | 0(1) |
|  |  | continuous variable | cardiovascular mortality | 0.287 | 60.4% | 1(0) |
|  |  | binary categorical variable | cardiovascular mortality | U | 53.0% | 0(1) |
|  |  | continuous variable | cardiovascular event | U | 75.2% | 1(0) |
| Kazemian^41^ | GNRI in patients who underwent TAVI |  | 1-year all-cause mortality | 0.080 | 96.4% | 0(1) |
| Li^73^ | GNRI in PC |  | OS | 0.118 | 80.9% | 1(1) |
|  |  |  | PFS | U | 79.5% | 0(0) |
| Yu^74^ | GNRI in HMs |  | OS | 0.043 | 67.9% | 0(0) |
|  |  |  | PFS | 0.014 | 86.3% | 0(1) |
|  |  |  | OS in lymphoma | 0.058 | 72.5% | 0(0) |
| Liu^75^ | GNRI in solid cancer |  | POCs | 0.858 | 25.0% | 0(1) |
|  |  |  | CD grades ≥2 | 0.360 | 14.1% | 0(0) |
|  |  |  | infection | 0.868 | 49.7% | 0(0) |
|  |  |  | ileus | 0.184 | 0.0% | 0(0) |
|  |  |  | leakage | 0.798 | 0.0% | 0(0) |
|  |  |  | respiratory complications | 0.270 | 68.4% | 0(0) |
| Luan^76^ | PNI in DLBCL |  | OS | 0.389 | 41.1% | 0(0) |
|  |  |  | PFS | 0.507 | 38.9% | 0(0) |
| Jiang^77^ | PNI in SCLC |  | OS | 0.054 | 66.8% | 0(0) |
|  |  |  | PFS | 0.283 | 63.3% | 0(1) |
| Ren^78^ | PNI in GIST |  | RFS, univariate analysis | 0.751 | 0.0% | 0(1) |
|  |  |  | RFS, multivariate analysis | 0.907 | 38.2% | 0(0) |
| Kang^79^ | PNI in GIST |  | RFS | 0.088 | 0.0% | 0(0) |
| Li^80^ | PNI in GIST |  | RFS | 0.998 | 37.5% | 0(0) |
| Hu^81^ | PNI in NSCLC |  | OS | 0.398 | 45.3% | 1(1) |
| Wang^82^ | PNI in NSCLC |  | OS | 0.001 | 81.0% | 1(1) |
|  |  |  | DFS/RFS | 0.068 | 90.45% | 0(1) |
|  |  |  | PFS | 0.715 | 0.0% | 0(0) |
|  | PNI in SCLC |  | OS | 0.939 | 85.9% | 0(0) |
| ZHANG^83^ | PNI in LC |  | PFS | 0.597 | 85.2% | 0(0) |
|  |  |  | OS | 0.584 | 94.9% | 1(1) |
|  | PNI in SCLC |  | PFS | U | 73.2% | 0(0) |
|  |  |  | OS | 0.866 | 90.9% | 0(1) |
|  | PNI in NSCLC |  | PFS | 0.646 | 87.7% | 0(0) |
|  |  |  | OS | 0.951 | 94.6% | 0(0) |
| Wang^84^ | PNI in glioma |  | OS | 0.345 | 0.0% | 0(0) |
| Liu^85^ | PNI in glioma |  | OS | 0.205 | 84.5% | 0(1) |
| Hung^86^ | PNI in glioma |  | favorable OS | 0.760 | 25.3% | 0(0) |
| Li^87^ | PNI in GC |  | OS | 0.002 | 49.3% | 1(1) |
|  |  |  | CSS | 0.367 | 0.0% | 0(0) |
|  |  |  | RFS | 0.007 | 80.6% | 0(0) |
|  |  |  | POCs | 0.322 | 61.7% | 0(1) |
| Yang^88^ | Low PNI in GC |  | OS | 0.140 | 6.9% | 0(0) |
|  |  |  | POCs | 0.594 | 35.4% | 0(0) |
| Lv^89^ | PNI in BTC |  | OS | 0.160 | 0.0% | 0(0) |
| Qi^90^ | PNI in UCs |  | OS | 0.005 | 0.0% | 0(0) |
|  |  |  | DFS/RFS/PFS | 0.110 | 0.0% | 0(0) |
|  |  |  | CSS/DSS | 0.632 | 40.7% | 0(0) |
| Sun^91^ | the low-PNI and high-PNI groups in CRC |  | OS | 0.000 | 87.6% | 1(1) |
|  |  |  | serious POCs | 0.048 | 0.0% | 0(0) |
| Yang^92^ | Low PNI in CRC |  | OS | 0.002 | 72.1% | 1(1) |
|  |  |  | CSS | 0.874 | 0.0% | 0(0) |
| Li^93^ | PNI in PC |  | OS | 0.201 | 25.2% | 0(0) |
| Liu^1^ | PNI |  | mortality after hip fracture | 0.289 | 70.8% | 0(0) |
| Hu^2^ | Low PNI in HF |  | all-cause mortality | 0.011 | 94.7% | 1(1) |
| Ni^39^ | PNI in HF |  | mortality | 0.990 | 88.0% | 0(0) |
| Zhang^94^ | PNI in HF | the lowest vs the highest | long-term all-cause mortality | 0.117 | 76.5% | 0(1) |
|  |  |  | long-term combined endpoint of mortality and re- hospitalization | 0.763 | 6.0% | 0(1) |
| Chen^95^ | PNI in HF | lower vs. higher | all-cause mortality | 0.202 | 70.4% | 0(0) |
|  |  |  | MACEs | 0.125 | 64.0% | 0(1) |
| Osório^4^ | PNI in patients hospitalized with decompensated HF |  | all-cause mortality | U | 88.9% | 0(1) |
| Chang^96^ | PNI in ACS |  | all-cause mortality | 0.659 | 88.9% | 1(1) |
|  |  |  | MACEs | 0.045 | 21.4% | 0(0) |
|  |  |  | MACCEs | 0.325 | 0.0% | 1(0) |
| Shi^97^ | PNI in HNNs |  | OS | 0.003 | 22.8% | 1(1) |
|  |  |  | DMFS | 0.805 | 0.0% | 0(0) |
|  |  |  | PFS | 0.074 | 0.0% | 0(0) |
| Luan^98^ | PNI in HNC |  | OS | 0.000 | 41.7% | 1(1) |
|  |  |  | PFS | 0.598 | 25.3% | 0(0) |
|  |  |  | DFS | 0.138 | 83.8% | 0(1) |
|  |  |  | DSS | 0.005 | 0.0% | 0(1) |
|  |  |  | DMFS | 0.534 | 0.0% | 0(0) |
| Peng^40^ | PNI in glioblastoma | univariate analysis | OS | 0.732 | 77.6% | 0(1) |
|  |  | multivariate analysis | OS | 0.362 | 24.9% | 0(0) |
|  |  | univariate analysis | PFS | 0.181 | 0.0% | 0(0) |
|  |  | multivariate analysis | PFS | U | 0.0% | 0(0) |
| Kazemian^41^ | PNI in patients who underwent TAVI |  | 1-year all-cause mortality | 0.879 | 2.0% | 1(0) |
| Peng^42^ | PNI in BC |  | OS | 0.734 | 0.0% | 0(0) |
|  |  |  | DFS | 0.157 | 87.5% | 0(1) |
| Hu^99^ | PNI in BC | low baseline PNI | OS | 0.952 | 0.0% | 0(0) |
|  |  | decreased baseline PNI | DFS | 0.269 | 84.0% | 0(1) |
| Meng^100^ | PNI in UTUC |  | OS | 0.016 | 0.0% | 0(1) |
|  |  |  | DFS/RFS/PFS | 0.939 | 0.0% | 0(0) |
|  |  |  | CSS/DSS | 0.967 | 0.0% | 0(0) |
| Dai^101^ | PNI in OC |  | OS | 0.225 | 75.0% | 0(0) |
|  |  |  | PFS | 0.400 | 20.0% | 0(0) |
| Tan^102^ | PNI in OC |  | OS | 0.100 | 87.3% | 0(0) |
|  |  |  | PFS | 0.044 | 86.0% | 0(0) |
|  |  |  | CSS | U | 0.0% | 0(0) |
| Fan^103^ | PNI in HCC |  | OS | 0.089 | 36.1% | 0(0) |
|  |  |  | RFS | 0.996 | 0.0% | 0(0) |
| Man^104^ | PNI in HCC |  | OS | 0.000 | 90.4% | 1(1) |
|  |  |  | DFS | 0.014 | 90.4% | 0(0) |
|  |  |  | recurrence | 0.160 | 57.1% | 0(1) |
| Liao^105^ | PNI in EC |  | OS | 0.020 | 54.1% | 0(0) |
|  |  |  | CSS | 0.028 | 38.8% | 0(0) |
|  |  |  | RFS | U | 0.0% | 0(0) |
| Hao^106^ | PNI in EC |  | OS | 0.118 | 82.7% | 1(1) |
|  |  |  | complications | 0.218 | 76.3% | 0(0) |
| Xue^107^ | PNI in EC |  | lymph node metastases | 0.296 | 79.6% | 1(1) |
|  |  |  | CSS | U | 65.6% | 0(1) |
|  |  |  | OS | 0.021 | 69.8% | 1(1) |
| Li^108^ | PNI in ESCC |  | OS | 0.091 | 32.7% | 0(1) |
|  |  |  | CSS | U | 90.5% | 0(1) |
|  |  |  | PFS | U | 0.0% | 0(0) |
| Mao^109^ | PNI in RCC |  | OS | 0.174 | 56.8% | 0(1) |
|  |  |  | PFS/DFS/RFS | 0.014 | 0.0% | 0(1) |
|  |  |  | CSS | 0.293 | 67.6% | 0(1) |
| Kim^110^ | PNI in RCC |  | OS/CSS | 0.001 | 8.8% | 0(0) |
|  |  |  | RFS/DFS | 0.758 | 17.3% | 0(0) |
| Xiong^111^ | PNI in RCC |  | OS | 0.042 | 45.7% | 1(1) |
|  |  |  | CSS | 0.019 | 87.6% | 1(1) |
|  |  |  | PFS/RFS/DFS | 0.003 | 0.0% | 0(0) |
| Peng^112^ | PNI in RCC |  | OS | 0.288 | 53.7% | 0(0) |
|  |  |  | CSS | 0.819 | 81.4% | 0(0) |
|  |  |  | DFS/PFS/RFS | 0.254 | 35.3% | 0(0) |
| Xue^113^ | PNI in RCC |  | OS | 0.000 | 84.0% | 1(1) |
|  |  |  | PFS | 0.000 | 83.4% | 0(0) |
|  |  |  | CSS | 0.000 | 85.8% | 1(1) |
|  |  |  | DFS | 0.002 | 77.9% | 0(0) |
|  |  |  | RFS | 0.205 | 54.7% | 0(1) |
| Wang^114^ | PNI in gynecological cancer | UVA | OS | 0.022 | 87.0% | 1(1) |
|  |  |  | PFS | 0.373 | 0.0% | 0(0) |
|  |  | MVA | OS | 0.024 | 89.1% | 1(1) |
|  |  |  | PFS | 0.293 | 0.0% | 0(0) |
| Zheng^115^ | PNI in PCa |  | OS | 0.003 | 74.9% | 0(1) |
|  |  |  | PFS | 0.061 | 51.5% | 0(1) |
| Dai^116^ | PNI in oral cancer |  | OS | 0.022 | 86.7% | 1(1) |
|  |  |  | DFS | 0.003 | 24.4% | 0(1) |
|  |  |  | CSS | U | 82.1% | 0(0) |
| Tang^117^ | PNI in NPC | UVA | OS | 0.085 | 0.0% | 0(0) |
|  |  |  | PFS | U | 67.8% | 1(1) |
|  |  |  | DMFS | 0.530 | 0.0% | 0(0) |
|  |  | MVA | OS | 0.103 | 0.0% | 0(0) |
|  |  |  | PFS | 0.431 | 7.3% | 0(1) |
|  |  |  | DMFS | 0.893 | 0.0% | 0(0) |
| Tu^118^ | PNI in NPC |  | OS | 0.010 | 0.0% | 0(1) |
|  |  |  | DMFS | 0.717 | 0.0% | 0(0) |
|  |  |  | PFS | 0.351 | 48.9% | 0(1) |
|  |  |  | LRRFS | 0.311 | 0.0% | 0(0) |
| Gao^119^ | PNI in NPC |  | OS | 0.028 | 9.0% | 0(0) |
|  |  |  | DMFS | 0.638 | 0.0% | 0(0) |
|  |  |  | PFS | 0.392 | 37.8% | 0(0) |
|  |  |  | LRFS | U | 0.0% | 0(0) |
|  |  |  | CSS | U | 26.6% | 0(0) |
| Zhang^120^ | PNI in CAD | PNI as a categorical variable | mortality | 0.013 | 94.7% | 1(1) |
|  |  |  | MACEs | 0.081 | 94.4% | 0(0) |
|  |  | PNI as a continuous variable | mortality | 0.379 | 88.6% | 1(0) |
|  |  |  | MACEs | 0.066 | 97.15 | 1(0) |
| Sun^121^ | PNI in cancer | POCs |  | U | 63.0% | 0(1) |
|  |  |  | CSS | U | 84.9% | 0(0) |
| Bullock^122^ | PNI in older adults with cancer (age ≥ 70) |  | OS | 0.177 | 65.2% | 0(0) |
| Guo^123^ | PNI in adult patients after spine surgery |  | POCs | 0.019 | 48.5% | 1(1) |
|  |  |  | POD | 0.007 | 37.8% | 0(1) |
|  |  |  | SSI | 0.412 | 0.0% | 0(0) |
| Jiao^46^ | PNI in bladder cancer |  | OS | 0.582 | 0.0% | 0(0) |
|  |  |  | RFS | 0.446 | 84.0% | 0(1) |
| Zhao^124^ | PNI in patients with PDAC who underwent curative resection |  | OS | 0.698 | 43.0% | 0(0) |
|  |  |  | RFS | U | 0.0% | 0(0) |
| Liu^125^ | PNI in PO-AKI |  | the risk of PO-AKI | 0.307 | 14.1% | 0(0) |
|  |  |  | PNI as Continuous variable | 0.209 | 87.5% | 1(0) |
|  | Low PNI | postoperative risk | infection | 0.226 | 0.0% | 0(0) |
|  |  |  | mortality | 0.529 | 0.0% | 0(0) |
|  |  |  | bleeding | 0.084 | 67.6% | 0(0) |
|  |  |  | stroke | 0.402 | 0.0% | 0(0) |
|  |  |  | intensive care unit stay | 0.098 | 85.4% | 1(1) |
|  |  |  | hospital stay | 0.353 | 80.7% | 0(1) |
| Li^126^ | PNI in advanced cancers receiving PD-­1/L1 inhibitors |  | OS | 0.516 | 33.0% | 0(0) |
|  |  |  | PFS | 0.674 | 0.0% | 0(0) |
|  |  |  | ORR | 0.584 | 0.0% | 0(0) |
|  |  |  | DCR | U | 0.0% | 0(0) |
| Ni^127^ | PNI in advanced-stage cancer patients treated with ICIs |  | PFS | 0.380 | 31.4% | 0(0) |
|  |  |  | DCR | 0.168 | 24.0% | 0(0) |
|  |  |  | ORR | 0.505 | 63.4% | 0(0) |
| Yan^128^ | PNI in in lung cancer patients receiving ICIs |  | OS | 0.753 | 53.5% | 0(0) |
|  |  |  | PFS | 0.147 | 30.4% | 0(0) |
| Xu^129^ | PNI in patients treated with ICIs |  | OS | 0.597 | 55.7% | 0(1) |
|  |  |  | PFS | 0.739 | 5.8% | 0(0) |
|  |  |  | ORR | 0.302 | 0.0% | 0(0) |
|  |  |  | DCR | 0.462 | 19.9% | 0(0) |
|  |  |  | adverse events | 0.725 | 65.7% | 0(0) |
| Zhang^130^ | PNI in patients with GIC treated with ICIs |  | OS | 0.003 | 34.4% | 0(0) |
|  |  |  | PFS | 0.027 | 22.0% | 0(0) |
|  |  |  | ORR | 0.080 | 0.0% | 0(0) |
|  |  |  | DCR | 0.239 | 0.0% | 0(0) |
| Zheng^131^ | PA in critically ill patients | as categorical variable | mortality | 0.021 | 0.0% | 0(0) |
|  |  | as continuous variable | mortality | 0.251 | 42.1% | 0(0) |
| Arab^132^ | PA in cancer |  | CSS | 0.667 | 0.0% | 0(0) |
| Fernández^133^ | PA in at-risk-pediatric patients |  | mortality | U | 0.0% | 0(0) |
|  |  |  | complications | U | 43.9% | 0(0) |
|  |  |  | length of hospital stay | U | 86.2% | 0(0) |
| Ahmadi^134^ | BMI in PD | BMI < 18.5 (underweight) | 1-year mortality | U | 0.0% | 0(0) |
|  |  |  | 2-year mortality | U | 45.3% | 1(0) |
|  |  |  | 3-5-year mortality | U | 45.6% | 0(0) |
| De Paola^135^ | BMI following MI | underweight | post-MI long term mortality | 0.274 | 78.8% | 0(0) |
|  |  | underweight | the risk of MI recurrence | U | 0.0% | 0(0) |
| Cao^136^ | BMI in COPD | underweight | the risk of MI recurrence | 0.051 | 65.9% | 0(0) |
| Simillis^137^ | BMI after colorectal cancer surgery | underweight，OS | colorectal cancer | 0.001 | 80.3% | 0(0) |
|  |  |  | only colon cancer | 0.107 | 68.7% | 0(0) |
|  |  |  | only rectal cancer | 0.102 | 53.0% | 0(0) |
| He^138^ | BMI in IPF | UVA | mortality | 0.018 | 45.0% | 1(0) |
|  |  | MVA | mortality | 0.037 | 61.8% | 1(0) |
|  |  | acute exacerbation | mortality | 0.195 | 55.9% | 1(0) |
|  |  |  | hospitalization | 0.331 | 71.0% | 1(0) |
| Abbreviations: ACS: acute coronary syndrome; BC: breast cancer; BMI: body mass index; BTC: biliary tract carcinoma; CAD: coronary artery disease; CD: Clavien-Dindo; CDc: Clavien-Dindo classification; CONUT: controlling nutritional status; COPD: Chronic Obstruct Pulmonary Disease; CRC: colorectal cancer; CSS: cancer-specific survival; DCR: disease control rate; DFS: disease-free survival; DSS: disease-specific survival; DLBCL: diffuse large B-cell lymphoma; DMFS: distant metastasis-free survival; EC: esophageal cancer; ESCC: esophageal squamous cell carcinoma; GC: gastric cancer; GI: gastrointestinal; GIC: gastrointestinal cancer; GIST: gastrointestinal stromal tumors; GLIM: global leadership initiative on malnutrition; GNRI: geriatric nutritional risk index; HCC: hepatocellular carcinoma; HF: heart failure; HMs: hematologic malignancies; HNC: head and neck cancer; HNNs: head and neck neoplasms; HPB: hepatopancreatobiliary; ICIs: immune checkpoint inhibitors; IPF: idiopathic pulmonary fibrosis; LC: lung cancer; LRFS: local recurrence free survival; MACCEs: major adverse cardiac and cerebrovascular events; MACEs: major adverse cardiovascular events; MACLEs: major adverse cardiovascular and leg events; mGPS: modified Glasgow Prognostic Score; MI: myocardial infarction; MNA, mini-nutritional assessment; MNA-SF, mini-nutritional assessment short-form; NPC: nasopharyngeal carcinoma; NPS: Naples prognostic score; NRS 2002: nutritional risk screening 2002; NSCLC: non-small cell lung cancer; NSTs: nutritional screening tools; MVA: multivariate analysis; OC: ovarian cancer; ORR: objective regression rate; OS: overall survival; PA: phase angle; PAD: peripheral artery disease; PC: pancreatic cancer; PCa: prostate cancer; PD: peritoneal dialysis; PDAC: pancreatic ductal adenocarcinoma; PFS: progression-free survival; PNI: prognostic nutritional index; PO-AKI: postoperative acute kidney injury; POCs: postoperative complications; POD: postoperative delirium; PD-­1/L1: programmed death-­1/programmed death-­ligand 1; PDAC: pancreatic ductal adenocarcinoma; RCC: renal cell carcinoma; RFS: relapse-free survival; SCLC: small cell lung cancer; SGA, subjective global assessment; SSI: surgical site infection; TAVI: transcatheter aortic valve implantation; UCs: urological cancers; UTUC: upper tract urothelial carcinoma; UVA: univariate analysis. | | | | | | |

**Reference:**

1. Liu N, Lv L, Jiao J, Zhang Y, Zuo XL. Association between nutritional indices and mortality after hip fracture: a systematic review and meta-analysis. *Eur Rev Med Pharmacol Sci*. Mar 2023;27(6):2297-2304. doi:10.26355/eurrev_202303_31763

2. Hu Y, Yang H, Zhou Y, et al. Prediction of all-cause mortality with malnutrition assessed by nutritional screening and assessment tools in patients with heart failure：a systematic review. *Nutr Metab Cardiovasc Dis*. Jun 2022;32(6):1361-1374. doi:10.1016/j.numecd.2022.03.009

3. Lin H, Zhang H, Lin Z, Li X, Kong X, Sun G. Review of nutritional screening and assessment tools and clinical outcomes in heart failure. *Heart Fail Rev*. Sep 2016;21(5):549-65. doi:10.1007/s10741-016-9540-0

4. Osório AF, Ribeiro ÉCT, Parahiba SM, Forte GC, Clausell NO, Souza GC. Prognostic value of nutritional screening tools in hospitalized patients with decompensated heart failure: A systematic review and meta-analysis. *Nutr Res*. Dec 2023;120:1-19. doi:10.1016/j.nutres.2023.09.009

5. Peng DD, Zong KZ, Yang H, et al. Malnutrition diagnosed by the Global Leadership Initiative on Malnutrition criteria predicting survival and clinical outcomes of patients with cancer: A systematic review and meta-analysis. *Front Nutr*. Dec 2022;9:1053165. 1053165. doi:10.3389/fnut.2022.1053165

6. Matsui R, Rifu K, Watanabe J, Inaki N, Fukunaga T. Impact of malnutrition as defined by the GLIM criteria on treatment outcomes in patients with cancer: A systematic review and meta-analysis. *Clin Nutr*. May 2023;42(5):615-624. doi:10.1016/j.clnu.2023.02.019

7. Xu J, Jie Y, Sun Y, Gong D, Fan Y. Association of Global Leadership Initiative on Malnutrition with survival outcomes in patients with cancer: A systematic review and meta-analysis. *Clin Nutr*. Sep 2022;41(9):1874-1880. doi:10.1016/j.clnu.2022.07.007

8. Yin L, Chong F, Huo Z, Li N, Liu J, Xu H. GLIM-defined malnutrition and overall survival in cancer patients: A meta-analysis. *JPEN J Parenter Enteral Nutr*. Feb 2023;47(2):207-219. doi:10.1002/jpen.2463

9. Lidoriki I, Frountzas M, Mela E, et al. The Prognostic Role of GLIM Criteria in Postoperative Outcomes after Upper Gastrointestinal Cancer Surgery: A Meta-Analysis of Observational Studies. *Nutr Cancer*. 2023;75(2):640-651. doi:10.1080/01635581.2022.2146144

10. Sun Z, Kong XJ, Jing X, Deng RJ, Tian ZB. Nutritional Risk Screening 2002 as a Predictor of Postoperative Outcomes in Patients Undergoing Abdominal Surgery: A Systematic Review and Meta-Analysis of Prospective Cohort Studies. *PLoS One*. 2015;10(7):e0132857. doi:10.1371/journal.pone.0132857

11. Zang Y, Xu W, Qiu Y, Gong D, Fan Y. Association between Risk of Malnutrition Defined by the Nutritional Risk Screening 2002 and Postoperative Complications and Overall Survival in Patients with Cancer: A Meta-Analysis. *Nutr Cancer*. 2023;75(8):1600-1609. doi:10.1080/01635581.2023.2227402

12. Chen FM, Xie C, Ren K, Xu XM. Prognostic Value of the Naples Prognostic Score in Patients with Gastrointestinal Cancers: A Meta-Analysis. *Nutr Cancer*. Aug 2023;75(7):1520-1530. doi:10.1080/01635581.2023.2212426

13. Wang YS, Niu L, Shi WX, Li XY, Shen L. Naples prognostic score as a predictor of outcomes in lung cancer: a systematic review and meta-analysis. *Eur Rev Med Pharmacol Sci*. Sep 2023;27(17):8144-8153. doi:10.26355/eurrev_202309_33574

14. Wu D, Wang X, Shi G, Sun H, Ge G. Prognostic and clinical significance of modified glasgow prognostic score in pancreatic cancer: a meta-analysis of 4,629 patients. *Aging (Albany NY)*. Jan 6 2021;13(1):1410-1421. doi:10.18632/aging.202357

15. Nie D, Zhang LP, Wang CY, Guo Q, Mao XG. A high Glasgow prognostic score (GPS) or modified Glasgow prognostic score (mGPS) predicts poor prognosis in gynecologic cancers: a systematic review and meta-analysis. *Arch Gynecol Obstet*. Jun 2020;301(6):1543-1551. doi:10.1007/s00404-020-05581-8

16. Hu X, Wang Y, Yang WX, Dou WC, Shao YX, Li X. Modified Glasgow prognostic score as a prognostic factor for renal cell carcinomas: a systematic review and meta-analysis. *Cancer Manag Res*. 2019;11(4):6163-6173. doi:10.2147/CMAR.S208839

17. Zhou Y, Liu Z, Cheng Y, Li J, Fu W. Prognostic value of the modified Glasgow prognostic score in biliary tract cancer patients: a systematic review and meta-analysis. *J Gastrointest Surg*. Jan 2024;28(4):559-565. doi:10.1016/j.gassur.2024.01.023

18. Zhang X, Tang T, Pang L, et al. Malnutrition and overall survival in older adults with cancer: A systematic review and meta-analysis. *J Geriatr Oncol*. Nov 2019;10(6):874-883. doi:10.1016/j.jgo.2019.03.002

19. Ma XF, Zou WH, Sun Y. Prognostic Value of Pretreatment Controlling Nutritional Status Score for Patients With Pancreatic Cancer: A Meta-Analysis. *Front Oncol*. Jan 2022;11:770894. 770894. doi:10.3389/fonc.2021.770894

20. Niu XH, Zhu Z, Bao J. Prognostic significance of pretreatment controlling nutritional status score in urological cancers: a systematic review and meta-analysis. *Cancer Cell Int.* Feb 2021;21(1):126. 126. doi:10.1186/s12935-021-01813-2

21. Liu H, Yang XC, Liu DC, Tong C, Wen W, Chen RH. Clinical significance of the controlling nutritional status (CONUT) score in gastric cancer patients: A meta-analysis of 9,764 participants. *Front Nutr*. Apr 2023;10:1156006. 1156006. doi:10.3389/fnut.2023.1156006

22. Yin J, Qu J, Liang XX, Wang MM. Prognostic significance of controlling nutritional status score for patients with gastric cancer: A systematic review and meta-analysis. *Exp Ther Med*. May 2023;25(5):202. 202. doi:10.3892/etm.2023.11901

23. Takagi K, Domagala P, Polak WG, Buettner S, Wijnhoven BPL, Ijzermans JNM. Prognostic significance of the controlling nutritional status (CONUT) score in patients undergoing gastrectomy for gastric cancer: a systematic review and meta-analysis. *BMC Surg*. Sep 5 2019;19(1):129. doi:10.1186/s12893-019-0593-6

24. Liu ZR, Zhou HG, Zhou Y, Yu ML, Cheng YL, Li J. Prognostic impact of the Controlling Nutritional Status Score in patients with biliary tract cancer: a systematic review and meta-analysis. *Front Oncol*. Aug 2023;13:1240008. 1240008. doi:10.3389/fonc.2023.1240008

25. Jiang H, Wang Z. Prognostic role of the controlling nutritional status (CONUT) score in patients with biliary tract cancer: a meta-analysis. *Ann Med*. 2023;55(2):2261461. doi:10.1080/07853890.2023.2261461

26. Chen J, Song P, Peng Z, et al. The Controlling Nutritional Status (CONUT) Score and Prognosis in Malignant Tumors: A Systematic Review and Meta-Analysis. *Nutr Cancer*. 2022;74(9):3146-3163. doi:10.1080/01635581.2022.2059091

27. Chen J, Cao D, Peng Z, et al. The prognostic value of the Controlling Nutritional Status score on patients undergoing nephrectomy for upper tract urothelial carcinoma or renal cell carcinoma: a systematic review and meta-analysis. *Br J Nutr*. Jul 28 2022;128(2):217-224. doi:10.1017/s0007114521002889

28. Xue W, Hu X, Zhang Y. The Association of Controlling Nutritional Status (CONUT) Score with Survival in Patients with Surgically Treated Renal Cell Carcinoma and Upper Tract Urothelial Carcinoma: A Systematic Review and Meta-Analysis. *Nutr Cancer*. 2022;74(6):1907-1916. doi:10.1080/01635581.2021.1974894

29. Peng L, Meng C, Li J, et al. The prognostic significance of controlling nutritional status (CONUT) score for surgically treated renal cell cancer and upper urinary tract urothelial cancer: a systematic review and meta-analysis. *Eur J Clin Nutr*. Jun 2022;76(6):801-810. doi:10.1038/s41430-021-01014-0

30. Shao J, Li J, Zhang XL, Wang G. Prognostic Significance of the Preoperative Controlled Nutritional Status Score in Lung Cancer Patients Undergoing Surgical Resection. *Nutr Cancer*. 2021;73(11-12):2211-2218. doi:10.1080/01635581.2020.1850814

31. Zhang C, Li XK, Cong ZZ, et al. Controlling nutritional status is a prognostic factor for patients with lung cancer: a systematic review and meta-analysis. *Ann Palliat Med*. Apr 2021;10(4):3896-3905. doi:10.21037/apm-20-2328

32. Takagi K, Buettner S, Ijzermans JNM. Prognostic significance of the controlling nutritional status (CONUT) score in patients with colorectal cancer: A systematic review and meta-analysis. *Int J Surg*. Jun 2020;78:91-96. doi:10.1016/j.ijsu.2020.04.046

33. Takagi K, Buettner S, Ijzermans JNM, Wijnhoven BPL. Systematic Review on the Controlling Nutritional Status (CONUT) Score in Patients Undergoing Esophagectomy for Esophageal Cancer. *Anticancer Res*. Oct 2020;40(10):5343-5349. doi:10.21873/anticanres.14541

34. Lv J, Chen P, Wu J, Hu C. Prognostic value of pretreatment Controlling Nutritional Status score in esophageal cancer: a meta-analysis. *Pathol Oncol Res*. 2023;29:1611221. doi:10.3389/pore.2023.1611221

35. Niu Z, Yan B. Prognostic and clinicopathological impacts of Controlling Nutritional Status (CONUT) score on patients with gynecological cancer: a meta-analysis. *Nutr J*. Jul 8 2023;22(1):33. doi:10.1186/s12937-023-00863-8

36. Lu C, Chen Q, Fei L, Wang J, Wang C, Yu L. Prognostic impact of the controlling nutritional status score in patients with hematologic malignancies: A systematic review and meta-analysis. *Front Immunol*. 2022;13:952802. doi:10.3389/fimmu.2022.952802

37. Peng J, Hao Y, Rao B, Cao Y. Prognostic impact of the pre-treatment controlling nutritional status score in patients with non-small cell lung cancer: A meta-analysis. *Medicine (Baltimore)*. Jul 2 2021;100(26):e26488. doi:10.1097/md.0000000000026488

38. Li HY, Zhou P, Zhao YK, Ni HC, Luo XP, Li J. Prediction of all-cause mortality with malnutrition assessed by controlling nutritional status score in patients with heart failure: a systematic review and meta-analysis. *Public Health Nutr*. Jul 2022;25(7):1799-1806. Pii s1368980021002470. doi:10.1017/S1368980021002470

39. Ni J, Fang Y, Zhang J, Chen X. Predicting prognosis of heart failure using common malnutrition assessment tools: A systematic review and meta-analysis. *Scott Med J*. Nov 2022;67(4):157-170. doi:10.1177/00369330221122300

40. Peng J, Li XY, Huang MS, et al. Prognostic value of prognostic nutritional index score and controlling nutritional status score in patients with glioblastoma: A comprehensive meta-analysis. *Front Oncol*. Feb 16 2023;13:1117764. 1117764. doi:10.3389/fonc.2023.1117764

41. Kazemian S, Tavolinejad H, Rashedi S, Yarahmadi P, Farrokhpour H, Kolte D. Meta-Analysis on the Association Between Nutritional Status and Outcomes After Transcatheter Aortic Valve Implantation. *Am J Cardiol*. Jan 1 2023;186:109-116. doi:10.1016/j.amjcard.2022.10.016

42. Peng P, Chen L, Shen Q, Xu Z, Ding X. Prognostic Nutritional Index (PNI) and Controlling Nutritional Status (CONUT) score for predicting outcomes of breast cancer: A systematic review and meta-analysis. *Pak J Med Sci*. Sep-Oct 2023;39(5):1535-1541. doi:10.12669/pjms.39.5.7781

43. Takagi K, Domagala P, Polak WG, Buettner S, Ijzermans JNM. The Controlling Nutritional Status Score and Postoperative Complication Risk in Gastrointestinal and Hepatopancreatobiliary Surgical Oncology: A Systematic Review and Meta-Analysis. *Ann Nutr Metab*. 2019;74(4):303-312. doi:10.1159/000500233

44. Zhang Y, Zhang X. Controlling nutritional status score, a promising prognostic marker in patients with gastrointestinal cancers after surgery: A systematic review and meta-analysis. *Int J Surg*. Jul 2018;55:39-45. doi:10.1016/j.ijsu.2018.05.018

45. Li L, Shou L. Prognostic and clinicopathological significance of the Controlling Nutritional Status (CONUT) score in patients with lymphoma: a meta-analysis. *BMJ Open*. Mar 7 2024;14(3):e078320. doi:10.1136/bmjopen-2023-078320

46. Jiao H, Wang L, Zhou X, Wu J, Li T. Prognostic Ability of Nutritional Indices for Outcomes of Bladder Cancer: A Systematic Review and Meta-Analysis. *Urol Int*. Aug 29 2023;107(9):886-894. doi:10.1159/000531884

47. Huang LJ, He RL, Sun XJ, Lv J, Chen SX. Association of Controlling Nutritional Status Score With Adverse Outcomes in Patients With Coronary Artery Disease: A Systematic Review and Meta-Analysis. *Angiology*. Feb 2023;74(2):149-158. 00033197221094409. doi:10.1177/00033197221094409

48. Yan CK, Xie YY, Hua YQ, et al. Prognostic value of geriatric nutritional risk index in patients with diffuse large B-cell lymphoma: a meta-analysis. *Clin Transl Oncol*. Jul 12 2023;26(2):515-523. doi:10.1007/s12094-023-03271-w

49. Cao D, Zhang ZX. Prognostic and clinicopathological role of geriatric nutritional risk index in patients with diffuse large B-cell lymphoma: A meta-analysis. *Front Oncol*. Mar 30 2023;13:1169749. 1169749. doi:10.3389/fonc.2023.1169749

50. Shen F, Ma Y, Guo W, Li F. Prognostic Value of Geriatric Nutritional Risk Index for Patients with Non-Small Cell Lung Cancer: A Systematic Review and Meta-Analysis. *Lung*. Oct 2022;200(5):661-669. doi:10.1007/s00408-022-00567-6

51. Yang M, Liu Z, Li G, et al. Geriatric Nutritional Risk Index as a Prognostic Factor of Patients with Non-Small Cell Lung Cancer: A Meta-Analysis. *Horm Metab Res*. Sep 2022;54(9):604-612. doi:10.1055/a-1903-1943

52. Yiu CY, Liu CC, Wu JY, et al. Efficacy of the Geriatric Nutritional Risk Index for Predicting Overall Survival in Patients with Head and Neck Cancer: A Meta-Analysis. Review. *Nutrients*. 2023;15(20):4348. doi:10.3390/nu15204348

53. Mao Y, Lan J. Prognostic value of the geriatric nutritional index in colorectal cancer patients undergoing surgical intervention: A systematic review and meta-analysis. *Front Oncol*. 2022;12:1066417. doi:10.3389/fonc.2022.1066417

54. Yuan F, Yuan Q, Hu J, An J. Prognostic Role of Pretreatment Geriatric Nutritional Risk Index in Colorectal Cancer Patients: A Meta-Analysis. *Nutr Cancer*. 2023;75(1):276-285. doi:10.1080/01635581.2022.2109692

55. Xu J, Sun Y, Gong D, Fan Y. Predictive Value of Geriatric Nutritional Risk Index in Patients with Colorectal Cancer: A Meta-Analysis. *Nutr Cancer*. 2023;75(1):24-32. doi:10.1080/01635581.2022.2115521

56. Zhao HM, Xu L, Tang P, Guo R. Geriatric Nutritional Risk Index and Survival of Patients With Colorectal Cancer: A Meta-Analysis. *Front Oncol*. Jun 30 2022;12:906711. 906711. doi:10.3389/fonc.2022.906711

57. Wu Q, Ye FG. Prognostic impact of geriatric nutritional risk index on patients with urological cancers: A meta-analysis. *Front Oncol*. Jan 11 2023;12:1077792. 1077792. doi:10.3389/fonc.2022.1077792

58. He L, Li Y, Qu LL, Zhang F. Prognostic and clinicopathological value of the geriatric nutritional risk index in gastric cancer: A meta-analysis of 5,834 patients. *Front Surg*. Jan 6 2023;9:1087298. 1087298. doi:10.3389/fsurg.2022.1087298

59. Lu W, Shen J, Zou DH, Li P, Liu XC, Jian Y. Predictive role of preoperative geriatric nutritional risk index for clinical outcomes in surgical gastric cancer patients: A meta-analysis. *Front Surg*. Nov 2 2022;9:1020482. 1020482. doi:10.3389/fsurg.2022.1020482

60. Zhang Q, Zhang L, Jin Q, et al. The Prognostic Value of the GNRI in Patients with Stomach Cancer Undergoing Surgery. *J Pers Med*. Jan 13 2023;13(1):155. doi:10.3390/jpm13010155

61. Wang HY, Li C, Yang RY, Jin J, Liu D, Li WM. Prognostic Value of the Geriatric Nutritional Risk Index in Non-Small Cell Lung Cancer Patients: A Systematic Review and Meta-Analysis. *Front Oncol*. Jan 18 2022;18:794862. 794862. doi:10.3389/fonc.2021.794862

62. Wang Y, Luo L, Li J, Wang Y, Che G, Xie X. Prognostic Value of Pretreatment Geriatric Nutrition Risk Index in Lung Cancer Patients: A Meta-Analysis. Article. *Nutr Cancer*. 2022;74(9):3164-3171. doi:10.1080/01635581.2022.2059093

63. Xie HL, Tang SY, Wei LS, Gan JL. Geriatric nutritional risk index as a predictor of complications and long-term outcomes in patients with gastrointestinal malignancy: a systematic review and meta-analysis. *Cancer Cell Int*. Oct 31 2020;20(1):530. 530. doi:10.1186/s12935-020-01628-7

64. Fan Y, He L, Zhou YJ, Man CF. Predictive Value of Geriatric Nutritional Risk Index in Patients With Coronary Artery Disease: A Meta-Analysis. *Front Nutr*. Sep 29 2021;8:736884. 736884. doi:10.3389/fnut.2021.736884

65. Liu GD, Zou C, Jie Y, Wang P, Wang XY, Fan Y. Predictive Value of Geriatric Nutritional Risk Index in Patients With Lower Extremity Peripheral Artery Disease: A Meta-Analysis. *Front Nutr*. Jun 22 2022;9:903293. 903293. doi:10.3389/fnut.2022.903293

66. Zhou JF, Fang PH, Li XK, et al. Prognostic Value of Geriatric Nutritional Risk Index in Esophageal Carcinoma: A Systematic Review and Meta-Analysis. *Front Nutr*. Mar 25 2022;9:831283. 831283. doi:10.3389/fnut.2022.831283

67. Yang Q, Shen A, Chen X, Guo L, Peng H, Gao M. Clinical Significance of Nutrition and Inflammation in Esophageal Cancer Patients with Surgery: A Meta-Analysis. *Nutr Cancer*. 2022;74(9):3128-3139. doi:10.1080/01635581.2022.2056620

68. Yu J, Zhang W, Wang C, Hu Y. The Prognostic Value of Pretreatment Geriatric Nutritional Risk Index in Esophageal Cancer: A Meta-Analysis. *Nutr Cancer*. 2022;74(9):3202-3210. doi:10.1080/01635581.2022.2069273

69. Fan H, Ma W, Fu Y, Yi T, Tian J. Association of Geriatric Nutritional Risk Index with Survival Outcomes in Patients with Esophageal Squamous Cell Carcinoma: A Meta-Analysis. *Nutr Cancer*. 2022;74(8):2796-2802. doi:10.1080/01635581.2022.2028865

70. Li H, Cen K, Sun W, Feng B. Prognostic value of geriatric nutritional risk index in elderly patients with heart failure: a meta-analysis. *Aging Clin Exp Res*. Jun 2021;33(6):1477-1486. doi:10.1007/s40520-020-01656-3

71. Lv GY, An L, Sun DW. Geriatric Nutritional Risk Index Predicts Adverse Outcomes in Human Malignancy: A Meta-Analysis. *Dis Markers*. 2019;2019:4796598. doi:10.1155/2019/4796598

72. YUAN Na LF, LIU Huan-bing. GNRI can predict the outcomes of maintenance hemodialysis patients—a meta-analysis. *Chin J Blood Purif*. 2021;20(8):516-520+535. doi:10.3969/j.issn.1671-4091.2021.08.004

73. Li L, He J. Prognostic Role of Geriatric Nutritional Risk Index in Patients with Pancreatic Cancer: A Meta-Analysis. *Nutr Cancer*. 2023;75(7):1531-1540. doi:10.1080/01635581.2023.2209345

74. Yu Q, Tian M, Pi G, Jia Y, Jin X. Geriatric nutritional risk index as a predictor of prognosis in hematologic malignancies: a systematic review and meta-analysis. *Front Nutr*. 2023;10:1274592. doi:10.3389/fnut.2023.1274592

75. Liu W, Li M, Lian S, Hou X, Ling Y. Geriatric nutritional risk index as a predictor for postoperative complications in patients with solid cancers: a meta-analysis. *Front Oncol*. 2024;14:1266291. doi:10.3389/fonc.2024.1266291

76. Luan C, Wang F, Wei N, Chen B. Prognostic nutritional index and the prognosis of diffuse large b-cell lymphoma: A meta-analysis. Article. *Cancer Cell Int*. 2020;20(1):455. doi:10.1186/s12935-020-01535-x

77. Jiang AM, Zhao R, Liu N, et al. The prognostic value of pretreatment prognostic nutritional index in patients with small cell lung cancer and it's influencing factors: A meta-analysis of observational studies. Article. *J Thorac Dis*. 2020;12(10):5718-5728. doi:10.21037/jtd-20-1739

78. Ren W, Wang H, Xiang T, Liu G. Prognostic Role of Preoperative Onodera’s Prognostic Nutritional Index (OPNI) in Gastrointestinal Stromal Tumors: a Systematic Review and Meta-analysis. Review. *J Gastrointest Cancer*. 2023;54(3):731-738. doi:10.1007/s12029-022-00878-0

79. Kang N, Gu H, Ni Y, Wei X, Zheng S. Prognostic and clinicopathological significance of the Prognostic Nutritional Index in patients with gastrointestinal stromal tumours undergoing surgery: a meta-analysis. *BMJ Open*. Dec 1 2022;12(12):e064577. doi:10.1136/bmjopen-2022-064577

80. Li Z, Zhang D, Mo C, Zhu P, Fan X, Tang T. The prognostic significance of prognostic nutritional index in gastrointestinal stromal tumors: A systematic review and meta-analysis. *Medicine (Baltimore)*. Nov 25 2022;101(47):e32067. doi:10.1097/md.0000000000032067

81. Hu Y, Shen J, Liu R, et al. Prognostic value of pretreatment prognostic nutritional index in non-small cell lung cancer: A systematic review and meta-analysis. *Int J Biol Markers*. Nov 2018;33(4):372-378. doi:10.1177/1724600818799876

82. Wang Z, Wang Y, Zhang X, Zhang T. Pretreatment prognostic nutritional index as a prognostic factor in lung cancer: Review and meta-analysis. *Clin Chim Acta*. Nov 2018;486:303-310. doi:10.1016/j.cca.2018.08.030

83. Zhang Q, Bao J, Zhu ZY, Jin MX. Prognostic nutritional index as a prognostic factor in lung cancer patients receiving chemotherapy: a systematic review and meta-analysis. *Eur Rev Med Pharmacol Sci*. Sep 2021;25(18):5636-5652. doi:10.26355/eurrev_202109_26783

84. Wang DP, Kang K, Lin Q, Hai J. Prognostic Significance of Preoperative Systemic Cellular Inflammatory Markers in Gliomas: A Systematic Review and Meta-Analysis. *Clin Transl Sci*. Jan 2020;13(1):179-188. doi:10.1111/cts.12700

85. Liu M, Wang L. Prognostic significance of preoperative serum albumin, albumin-to-globulin ratio, and prognostic nutritional index for patients with glioma: A meta-analysis. *Medicine (Baltimore)*. Jul 2 2020;99(27):e20927. doi:10.1097/md.0000000000020927

86. Hung KC, Sun CK, Chang YP, et al. Association of prognostic nutritional index with prognostic outcomes in patients with glioma: a meta-analysis and systematic review. *Front Oncol*. 2023;13:1188292. doi:10.3389/fonc.2023.1188292

87. Li J, Xu R, Hu DM, Zhang Y, Gong TP, Wu XL. Prognostic Nutritional Index Predicts Outcomes of Patients after Gastrectomy for Cancer: A Systematic Review and Meta-Analysis of Nonrandomized Studies. *Nutr Cancer*. 2019;71(4):557-568. doi:10.1080/01635581.2019.1577986

88. Yang Y, Gao P, Song Y, et al. The prognostic nutritional index is a predictive indicator of prognosis and postoperative complications in gastric cancer: A meta-analysis. *Eur J Surg Oncol*. Aug 2016;42(8):1176-82. doi:10.1016/j.ejso.2016.05.029

89. Lv X, Zhang Z, Yuan W. Pretreatment Prognostic Nutritional Index (PNI) as a Prognostic Factor in Patients with Biliary Tract Cancer: A Meta-Analysis. *Nutr Cancer*. 2021;73(10):1872-1881. doi:10.1080/01635581.2020.1817955

90. Qi F, Zhou X, Wang Y, et al. Pre-treatment prognostic nutritional index may serve as a potential biomarker in urinary cancers: a systematic review and meta-analysis. *Cancer Cell Int*. 2018;18:207. doi:10.1186/s12935-018-0708-7

91. Sun G, Li Y, Peng Y, et al. Impact of the preoperative prognostic nutritional index on postoperative and survival outcomes in colorectal cancer patients who underwent primary tumor resection: a systematic review and meta-analysis. *Int J Colorectal Dis*. Apr 2019;34(4):681-689. doi:10.1007/s00384-019-03241-1

92. Yang Y, Gao P, Chen X, et al. Prognostic significance of preoperative prognostic nutritional index in colorectal cancer: results from a retrospective cohort study and a meta-analysis. *Oncotarget*. Sep 6 2016;7(36):58543-58552. doi:10.18632/oncotarget.10148

93. Li S, Tian G, Chen Z, Zhuang Y, Li G. Prognostic Role of the Prognostic Nutritional Index in Pancreatic Cancer: A Meta-analysis. *Nutr Cancer*. 2019;71(2):207-213. doi:10.1080/01635581.2018.1559930

94. Zhang X, Su Y. Low Prognostic Nutritional Index Predicts Adverse Outcomes in Patients With Heart Failure: A Systematic Review and Meta-analysis. *Angiology*. Feb 24 2023;75(4):305-313. doi:10.1177/00033197231159680

95. Chen MY, Wen JX, Lu MT, et al. Association Between Prognostic Nutritional Index and Prognosis in Patients With Heart Failure: A Meta-Analysis. *Front Cardiovasc Med*. 2022;9:918566. doi:10.3389/fcvm.2022.918566

96. Chang WT, Sun CK, Wu JY, et al. Association of prognostic nutritional index with long-term mortality in patients receiving percutaneous coronary intervention for acute coronary syndrome: a meta-analysis. *Sci Rep*. Aug 11 2023;13(1):13102. doi:10.1038/s41598-023-40312-4

97. Shi Y, Zhang Y, Niu Y, Chen Y, Kou C. Prognostic role of the prognostic nutritional index (PNI) in patients with head and neck neoplasms undergoing radiotherapy: A meta-analysis. *PLoS One*. 2021;16(9):e0257425. doi:10.1371/journal.pone.0257425

98. Luan CW, Tsai YT, Yang HY, Chen KY, Chen PH, Chou HH. Pretreatment prognostic nutritional index as a prognostic marker in head and neck cancer: a systematic review and meta-analysis. *Sci Rep*. Aug 24 2021;11(1):17117. doi:10.1038/s41598-021-96598-9

99. Hu G, Ding Q, Zhong K, Wang S, Wang S, Huang L. Low pretreatment prognostic nutritional index predicts poor survival in breast cancer patients: A meta-analysis. *PLoS One*. 2023;18(1):e0280669. doi:10.1371/journal.pone.0280669

100. Meng C, Gan L, Li K, et al. Prognostic nutritional index before surgical treatment may serve as a prognostic biomarker for patients with upper tract urothelial carcinoma: A systematic review and meta-analysis. *Front Nutr*. 2022;9:972034. doi:10.3389/fnut.2022.972034

101. Dai Y, Liu M, Lei L, Lu S. Prognostic significance of preoperative prognostic nutritional index in ovarian cancer: A systematic review and meta-analysis. *Medicine (Baltimore)*. Sep 18 2020;99(38):e21840. doi:10.1097/md.0000000000021840

102. Tan X, Chen H. The Prognostic Value of Prognostic Nutritional Index in Patients with Ovarian Cancer: A Systematic Review and Meta-Analysis. *Nutr Cancer*. 2023;75(1):73-81. doi:10.1080/01635581.2022.2104879

103. Fan X, Chen G, Li Y, et al. The Preoperative Prognostic Nutritional Index in Hepatocellular Carcinoma After Curative Hepatectomy: A Retrospective Cohort Study and Meta-Analysis. *J Invest Surg*. Aug 2021;34(8):826-833. doi:10.1080/08941939.2019.1698679

104. Man Z, Pang Q, Zhou L, et al. Prognostic significance of preoperative prognostic nutritional index in hepatocellular carcinoma: a meta-analysis. *HPB (Oxford)*. Oct 2018;20(10):888-895. doi:10.1016/j.hpb.2018.03.019

105. Liao G, Zhao Z, Yang H, Chen M, Li X. Can Prognostic Nutritional Index be a Prediction Factor in Esophageal Cancer?: A Meta-Analysis. *Nutr Cancer*. 2020;72(2):187-193. doi:10.1080/01635581.2019.1631859

106. Hao J, Chen C, Wan F, et al. Prognostic Value of Pre-Treatment Prognostic Nutritional Index in Esophageal Cancer: A Systematic Review and Meta-Analysis. *Front Oncol*. 2020;10:797. doi:10.3389/fonc.2020.00797

107. Xue Y, Zhou X, Xue L, Zhou R, Luo J. The role of pretreatment prognostic nutritional index in esophageal cancer: A meta-analysis. *J Cell Physiol*. Nov 2019;234(11):19655-19662. doi:10.1002/jcp.28565

108. Li P, Wang X, Lai Y, Zhou K, Tang Y, Che G. The prognostic value of pre-treatment prognostic nutritional index in esophageal squamous cell carcinoma: A meta-analysis. *Medicine (Baltimore)*. May 2019;98(22):e15280. doi:10.1097/md.0000000000015280

109. Mao C, Xu W, Ma W, Wang C, Guo Z, Yan J. Prognostic Value of Pretreatment Prognostic Nutritional Index in Patients With Renal Cell Carcinoma: A Meta-Analysis. *Front Oncol*. 2021;11:719941. doi:10.3389/fonc.2021.719941

110. Kim SI, Kim SJ, Kim SJ, Cho DS. Prognostic nutritional index and prognosis in renal cell carcinoma: A systematic review and meta-analysis. *Urol Oncol*. Oct 2021;39(10):623-630. doi:10.1016/j.urolonc.2021.05.028

111. Xiong SC, Hu X, Lia T, Wang YH, Li X. Prognostic Significance of Prognostic Nutritional Index in Patients with Renal Cell Carcinoma: A Meta-Analysis. *Nutr Cancer*. 2022;74(3):860-868. doi:10.1080/01635581.2021.1931702

112. Peng Q, Liu L, Li T, Lei C, Wan H. Prognostic impact of prognostic nutritional index on renal cell carcinoma: A meta-analysis of 7,629 patients. *PLoS One*. 2022;17(3):e0265119. doi:10.1371/journal.pone.0265119

113. Xue S, Zhao H, Zhang K, Zhang H, Wang W. Prognostic and Clinicopathological Correlations of Pretreatment Prognostic Nutritional Index in Renal Cell Carcinoma: A Meta-Analysis. *Urol Int*. 2022;106(6):567-580. doi:10.1159/000521353

114. Wang X, Wang Y. The prognostic nutritional index is prognostic factor of gynecological cancer: A systematic review and meta-analysis. *Int J Surg*. Jul 2019;67:79-86. doi:10.1016/j.ijsu.2019.05.018

115. Zheng Y, Wang K, Ou Y, et al. Prognostic value of a baseline prognostic nutritional index for patients with prostate cancer: a systematic review and meta-analysis. *Prostate Cancer Prostatic Dis*. Jun 30 2023;27(4):604-613. doi:10.1038/s41391-023-00689-9

116. Dai M, Sun Q. Prognostic and clinicopathological significance of prognostic nutritional index (PNI) in patients with oral cancer: a meta-analysis. *Aging (Albany NY)*. Mar 7 2023;15(5):1615-1627. doi:10.18632/aging.204576

117. Tang M, Jia Z, Zhang J. The prognostic role of prognostic nutritional index in nasopharyngeal carcinoma: A systematic review and meta-analysis. *Int J Clin Oncol*. Jan 2021;26(1):66-77. doi:10.1007/s10147-020-01791-x

118. Tu X, Ren J, Zhao Y. Prognostic value of prognostic nutritional index in nasopharyngeal carcinoma: A meta-analysis containing 4511 patients. *Oral Oncol*. Nov 2020;110:104991. doi:10.1016/j.oraloncology.2020.104991

119. Gao QL, Shi JG, Huang YD. Prognostic Significance of Pretreatment Prognostic Nutritional Index (PNI) in Patients with Nasopharyngeal Carcinoma: A Meta-Analysis. *Nutr Cancer*. 2021;73(9):1657-1667. doi:10.1080/01635581.2020.1810715

120. Zhang S, Wang H, Chen S, et al. Prognostic nutritional index and prognosis of patients with coronary artery disease: A systematic review and meta-analysis. *Front Nutr*. 2023;10:1114053. doi:10.3389/fnut.2023.1114053

121. Sun K, Chen S, Xu J, Li G, He Y. The prognostic significance of the prognostic nutritional index in cancer: a systematic review and meta-analysis. *J Cancer Res Clin Oncol*. Sep 2014;140(9):1537-49. doi:10.1007/s00432-014-1714-3

122. Bullock AF, Greenley SL, McKenzie GAG, Paton LW, Johnson MJ. Relationship between markers of malnutrition and clinical outcomes in older adults with cancer: systematic review, narrative synthesis and meta-analysis. *Eur J Clin Nutr*. Nov 2020;74(11):1519-1535. doi:10.1038/s41430-020-0629-0

123. Guo H, Yang L, Liu J, Yu X, Chen L, Huang Y. Prognostic Nutritional Index and the Risk of Postoperative Complications After Spine Surgery: A Meta-Analysis. *World Neurosurg*. Feb 19 2024;185:e572-e581. doi:10.1016/j.wneu.2024.02.077

124. Zhao P, Wu Z, Wang Z, Wu C, Huang X, Tian B. Prognostic role of the prognostic nutritional index in patients with pancreatic cancer who underwent curative resection without preoperative neoadjuvant treatment: A systematic review and meta-analysis. *Front Surg*. 2022;9:992641. doi:10.3389/fsurg.2022.992641

125. Liu CC, Liu PH, Chen HT, et al. Association of Preoperative Prognostic Nutritional Index with Risk of Postoperative Acute Kidney Injury: A Meta-Analysis of Observational Studies. *Nutrients*. Jun 28 2023;15(13):2929. doi:10.3390/nu15132929

126. Li P, Lai Y, Tian L, Zhou Q. The prognostic value of prognostic nutritional index in advanced cancer receiving PD-1/L1 inhibitors: A meta-analysis. *Cancer Med*. Aug 2022;11(16):3048-3056. doi:10.1002/cam4.4668

127. Ni L, Huang J, Ding J, et al. Prognostic Nutritional Index Predicts Response and Prognosis in Cancer Patients Treated With Immune Checkpoint Inhibitors: A Systematic Review and Meta-Analysis. *Front Nutr*. 2022;9:823087. doi:10.3389/fnut.2022.823087

128. Yan X, Wang J, Mao J, et al. Identification of prognostic nutritional index as a reliable prognostic indicator for advanced lung cancer patients receiving immune checkpoint inhibitors. *Front Nutr*. 2023;10:1213255. doi:10.3389/fnut.2023.1213255

129. Xu XT, Qian Y, Tian MX, et al. Predictive Impact of Prognostic Nutritional Index in Patients with Cancer Treated with Immune Checkpoint Inhibitors: A Systematic Review and Meta-Analysis. *Nutr Cancer*. 2023;75(6):1413-1426. doi:10.1080/01635581.2023.2203355

130. Zhang L, Ma W, Qiu Z, et al. Prognostic nutritional index as a prognostic biomarker for gastrointestinal cancer patients treated with immune checkpoint inhibitors. *Front Immunol*. 2023;14:1219929. doi:10.3389/fimmu.2023.1219929

131. Zheng WH, Zhao YH, Yao Y, Huang HB. Prognostic role of bioelectrical impedance phase angle for critically ill patients: A systemic review and meta-analysis. Review. *Front Med (Lausanne)*. 2023;9:1059747. doi:10.3389/fmed.2022.1059747

132. Arab A, Karimi E, Vingrys K, Shirani F. Is phase angle a valuable prognostic tool in cancer patients' survival? A systematic review and meta-analysis of available literature. *Clin Nutr*. May 2021;40(5):3182-3190. doi:10.1016/j.clnu.2021.01.027

133. Fernández-Jiménez R, Martín-Masot R, Cornejo-Pareja I, et al. Phase angle as a marker of outcome in hospitalized pediatric patients. A systematic review of the evidence (GRADE) with meta-analysis. *Rev Endocr Metab Disord*. Aug 2023;24(4):751-765. doi:10.1007/s11154-023-09817-1

134. Ahmadi SF, Zahmatkesh G, Streja E, et al. Association of body mass index with mortality in peritoneal dialysis patients: A systematic review and meta-analysis. Article. *Perit Dial Int*. 2016;36(3):315-325. doi:10.3747/pdi.2015.00052

135. De Paola L, Mehta A, Pana TA, et al. Body Mass Index and Mortality, Recurrence and Readmission after Myocardial Infarction: Systematic Review and Meta-Analysis. *J Clin Med*. May 5 2022;11(9)doi:10.3390/jcm11092581

136. Cao C, Wang R, Wang JM, Bunjhoo H, Xu YJ, Xiong WN. Body Mass Index and Mortality in Chronic Obstructive Pulmonary Disease: A Meta-Analysis. *PLOS ONE*. Aug 24 2012;7(8):e43892. e43892. doi:10.1371/journal.pone.0043892

137. Simillis C, Taylor B, Ahmad A, et al. A systematic review and meta-analysis assessing the impact of body mass index on long-term survival outcomes after surgery for colorectal cancer. *Eur J Cancer*. Sep 2022;172:237-251. doi:10.1016/j.ejca.2022.05.020

138. He X, Ji J, Liu C, et al. Body mass index and weight loss as risk factors for poor outcomes in patients with idiopathic pulmonary fibrosis: a systematic review and meta-analysis. *Ann Med*. Dec 2024;56(1):2311845. doi:10.1080/07853890.2024.2311845
