# Supplementary material for: Association between malnutrition diagnosed by different screening and assessment tools and clinical outcomes: an umbrella review
Source: Front Nutr. 2025 Oct 9;12:1676201. doi: 10.3389/fnut.2025.1676201 (PMC12545068; doi:10.3389/fnut.2025.1676201)
Supplement: Supplementary file 6 [file Table_6.PDF]

SPRINGER NATURE
Author Services

Editing Certificate

This document certifies that the manuscript

Association between malnutrition diagnosed by different screening and asscessment tools and clinical outcomes: an umbrella review

prepared by the authors

Zhinan Li, Yueying Lin, Yanmei Shi,Ting Yang, Liya An, Yuxing Qi, Pengcheng Zhang, Xingzong Huang, Xianming Sa, Yinlong Deng, Jian Hu, Guobin Liu*, Dali Sun

was edited for proper English language, grammar, punctuation, spelling, and overall style by one or more of the highly qualified English speaking editors at SNAS.

This certificate was issued on **February 10,2025** and may be verified on the SNAS website using the verification code **4E77-DA6D-F077-D274-99F9**.

Neither the research content nor the authors' intentions were altered in any way during the editing process. Documents receiving this certification should be English-ready for publication; however, the author has the ability to accept or reject our suggestions and changes. To verify the final SNAS edited version, please visit our verification page at secure.authorservices.springernature.com/certificate/verify.
If you have any questions or concerns about this edited document, please contact SNAS at support@as.springernature.com.

SNAS provides a range of editing, translation, and manuscript services for researchers and publishers around the world.
For more information about our company, services, and partner discounts, please visit authorservices.springernature.com.
